# Dual-hybrid intrusion detection system to detect False Data Injection in smart grids

**DOI:** 10.1371/journal.pone.0316536

**Published:** 2025-01-27

**Authors:** Saad Hammood Mohammed, Mandeep S. Jit Singh, Abdulmajeed Al-Jumaily, Mohammad Tariqul Islam, Md. Shabiul Islam, Abdulmajeed M. Alenezi, Mohamed S. Soliman

**Affiliations:** 1 Department of Electrical Electronic and Systems Engineering Faculty of Engineering and Built Environment, Universiti Kebangsaan Malaysia (UKM) Bangi, Selangor, Malaysia; 2 Department of Signal Theory and Communications Universidad Carlos III de Madrid, Leganés, Spain; 3 Faculty of Engineering (FOE) Multimedia University (MMU) Cyberjaya, Selangor, Malaysia; 4 Department of Electrical Engineering, Faculty of Engineering, Islamic University of Madinah, Madinah, Saudi Arabia; 5 Department of Electrical Engineering, College of Engineering, Taif University, Taif, Saudi Arabia; Cyprus International University Faculty of Engineering: Uluslararasi Kibris Universitesi Muhendislik Fakultesi, TÜRKIYE

## Abstract

Modernizing power systems into smart grids has introduced numerous benefits, including enhanced efficiency, reliability, and integration of renewable energy sources. However, this advancement has also increased vulnerability to cyber threats, particularly False Data Injection Attacks (FDIAs). Traditional Intrusion Detection Systems (IDS) often fall short in identifying sophisticated FDIAs due to their reliance on predefined rules and signatures. This paper addresses this gap by proposing a novel IDS that utilizes hybrid feature selection and deep learning classifiers to detect FDIAs in smart grids. The main objective is to enhance the accuracy and robustness of IDS in smart grids. The proposed methodology combines Particle Swarm Optimization (PSO) and Grey Wolf Optimization (GWO) for hybrid feature selection, ensuring the selection of the most relevant features for detecting FDIAs. Additionally, the IDS employs a hybrid deep learning classifier that integrates Convolutional Neural Networks (CNN) and Long Short-Term Memory (LSTM) networks to capture the smart grid data’s spatial and temporal features. The dataset used for evaluation, the Industrial Control System (ICS) Cyber Attack Dataset (Power System Dataset) consists of various FDIA scenarios simulated in a smart grid environment. Experimental results demonstrate that the proposed IDS framework significantly outperforms traditional methods. The hybrid feature selection effectively reduces the dimensionality of the dataset, improving computational efficiency and detection performance. The hybrid deep learning classifier performs better in key metrics, including accuracy, recall, precision, and F-measure. Precisely, the proposed approach attains higher accuracy by accurately identifying true positives and minimizing false negatives, ensuring the reliable operation of smart grids. Recall is enhanced by capturing critical features relevant to all attack types, while precision is improved by reducing false positives, leading to fewer unnecessary interventions. The F-measure balances recall and precision, indicating a robust and reliable detection system. This study presents a practical dual-hybrid IDS framework for detecting FDIAs in smart grids, addressing the limitations of existing IDS techniques. Future research should focus on integrating real-world smart grid data for validation, developing adaptive learning mechanisms, exploring other bio-inspired optimization algorithms, and addressing real-time processing and scalability challenges in large-scale deployments.

## 1 Introduction

**Fig 1:**
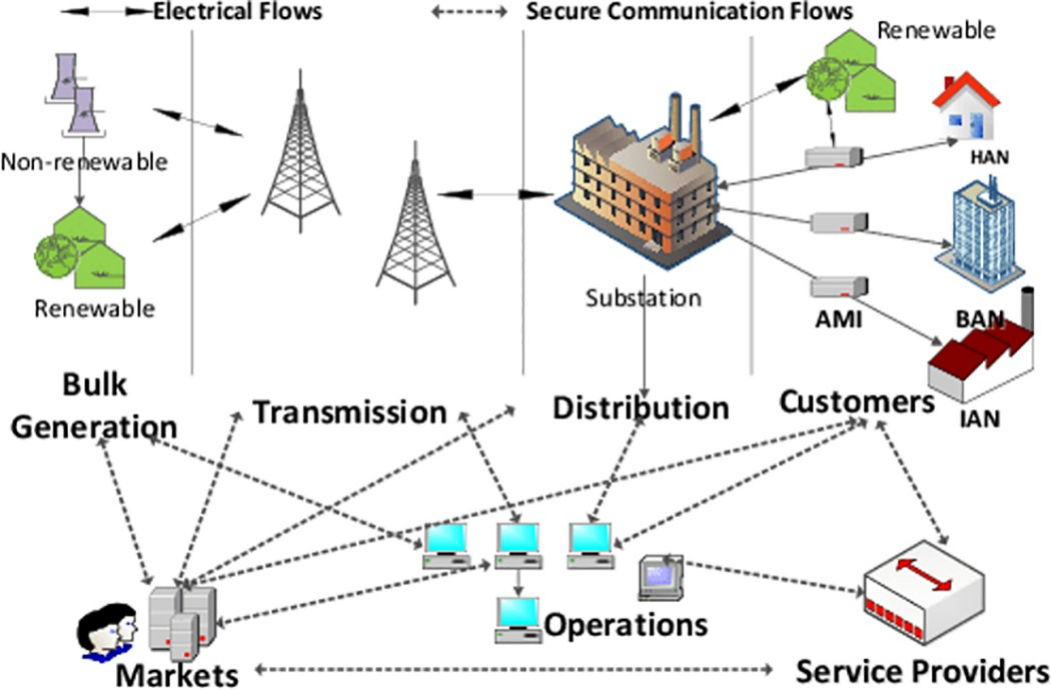
Conceptual model of a smart grid system. This figure illustrates the conceptual model of a smart grid system, highlighting key components and their interactions within the grid environment.

The smart grid is a complex electricity generation, transmission, distribution, and utilization system composed of end-use customers. It is characterized by distributed generators, renewable energy sources (e.g., wind, solar, biomass), energy storage facilities, and observability and controllability that extend throughout the interconnected portion of the power system. It also has critical interface capabilities with advanced communication networks. Therefore, the smart grid can answer various physical and cyber threats and failures. It enhances the power network with intelligent monitoring and control. Consequently, it is considered the technology of the next-generation power system. The conceptual model of a smart grid system is presented in Fig 1.

Modernizing power systems into smart grids has introduced many benefits, including enhanced efficiency, reliability, and integration of renewable energy sources [1]. However, these advancements have also opened new avenues for cyber threats, notably False Data Injection Attacks (FDIAs) [1–3]. FDIA is a critical concern because it involves maliciously injecting false data into the grid’s state estimation process, leading to incorrect operational decisions, economic losses, and even large-scale blackouts [1]. Therefore, developing robust mechanisms to detect and mitigate such attacks is paramount for the security and stability of smart grids [4, 5].

Intrusion Detection Systems (IDS) have emerged as a fundamental component in the defence against cyber-attacks in smart grids [6, 7]. Traditional IDS techniques, however, often fall short of effectively identifying complex and sophisticated FDIAs due to their reliance on predefined rules and signatures [6, 8, 9]. This limitation necessitates the exploration of advanced methodologies that can adapt to the dynamic nature of cyber threats in smart grids. Hybrid approaches, which combine different detection mechanisms and leverage various data features, have shown promise in enhancing the detection capabilities of IDS. Feature selection plays a crucial role in improving the performance of IDS by reducing the dimensionality of the data and enhancing the accuracy of detection algorithms [10]. A hybrid feature selection method, which integrates filter and wrapper techniques, can effectively select the most relevant features for detecting FDIAs [11]. This approach not only improves the efficiency of the IDS but also enhances its ability to generalize across different attack scenarios. The IDS can achieve higher detection rates and lower false alarm rates by focusing on the most informative features.

Deep learning classifiers have gained significant attention in intrusion detection due to their ability to learn and extract complex patterns from large datasets automatically [8, 9, 12]. However, relying on a single deep learning model may not be sufficient to capture the diverse characteristics of FDIAs [7]. A hybrid deep learning approach, which combines multiple classifiers, can provide a more comprehensive detection mechanism [11]. This method leverages the strengths of different deep learning models to improve overall detection performance and robustness against various types of FDIAs. As aforementioned, the Smart Grid faces a significant threat from sophisticated cyber-physical attacks known as FDIAs [13]. These attacks involve adversaries’ covert insertion of manipulated data, compromising essential smart grid data and obscuring the power system’s operational status, potentially leading to widespread operational failures [14]. While current cybersecurity solutions have shown effectiveness in simulations, there is a critical need for validation against real benchmark datasets. Most FDIA methodologies lack commercial-level datasets necessary for accurately assessing vulnerabilities, emphasizing the need for integrating real-world data to enhance countermeasure development. The complexity of the smart grid requires diverse cyber-physical benchmark datasets for thorough experimental validation, which is currently lacking in many studies.

Moreover, contemporary cybersecurity systems struggle with managing the vast and complex data volumes generated within networked environments, posing challenges in detecting sophisticated cyber threats like FDIAs. Traditional feature selection methods are insufficient in discerning critical data patterns, and there is a noticeable gap in leveraging advanced bio-inspired optimization algorithms for feature selection. Algorithms like Particle PSO [15] and GWO [10] offer potential improvements but remain underexplored in securing smart grids. Additionally, existing IDS optimization functions often prioritize a single performance metric, failing to address the broader spectrum of operational requirements. There is a need for multi-objective optimization functions to balance various performance criteria effectively. The rapid sophistication of FDIAs necessitates hybrid deep learning models combining CNNs and LSTM networks to enhance detection capabilities and adaptability.

Securing smart grids against FDIAs is critical due to the potential for severe real-world consequences. Smart grids form the backbone of modern power systems, enabling the integration of renewable energy sources and enhancing operational efficiency. However, their increased connectivity and reliance on digital communication have made them vulnerable to cyber threats. FDIAs, in particular, pose a unique risk by allowing attackers to inject misleading data into grid operations, leading to incorrect state estimations and faulty operational decisions. This can result in substantial economic losses due to disrupted power delivery, increased operational costs, and, in worst-case scenarios, large-scale blackouts impacting millions of users. Current IDS are often inadequate in combating such sophisticated attacks, primarily due to their dependence on rule-based or signature-based techniques. These traditional approaches struggle to adapt to the evolving nature of FDIAs, which can bypass static detection rules by mimicking legitimate data patterns. This limitation underscores the need for more advanced detection mechanisms. To address these challenges, this paper proposes an innovative IDS framework that enhances feature selection and leverages deep learning methodologies, thus improving detection accuracy and resilience against complex, adaptive cyber threats in smart grids. By integrating bio-inspired feature selection methods with a hybrid deep learning classifier, this paper provides a robust solution for securing smart grid infrastructures, ultimately contributing to the reliability and safety of critical power systems.

The core goal of this paper is to propose an efficient IDS in smart grids based on deep learning and bio-inspired feature selection. The following objectives are formulated to achieve the primary goal of this paper:

- To analyze the role of using a hybrid feature selection optimization (PSO and GWO) in enhancing the performance of IDS. This hybridized feature selection approach intends to meticulously identify and select the most significant essential features (i.e., feature subset) for improving the performance of IDS.
- To develop a multi-objective function (i.e., fitness function). This function is meticulously proposed to balance multiple criteria, which often compete with objectives such as maximization of accuracy, minimization of number of features, and enhancement of model robustness, including precision and recall.
- To validate an efficient hybrid deep learning model (Hybridizing CNN and LSTM) particularly tailored for detecting false data injection attacks. This hybrid deep learning model integrates the strengths of multiple deep learning architectures (i.e., CNN and LSTM), leveraging their unique features to improve detection performance.

### 1.1 Background and significance

Each component of the smart grid, supervised by advanced communication networks and computer systems, exchanges vital information that forms the basis of paramount actions [1]. Therefore, the smart grid considers control of the critical infrastructure, especially the safe and secure operation of this grid, as a primary goal. The goal is accomplished by creating defence zones with classical principles such as policy and privilege stability. It also ensures the system is always up-to-date to satisfy the required authenticity, integrity, and privacy requirements. Additionally, novel intrusion detection and early response techniques are employed [6]. This goal is to be satisfied through modern intrusion detection systems. These systems, which belong to the class of must not have false alarms to untrained personnel, are treated in the present paper. In particular, the combined approaches through hybrid feature selection, hybrid deep learning, and fine-tuning techniques are investigated in this manuscript.

## 2 Smart grids and False Data Injection Attacks

Smart grids are a term used to describe a modern electrical system that has digitally integrated advanced communications, new information technologies, and strategies that significantly improve energy conservation and efficiency, as well as minimize the costs of a smart grid project [8, 9, 16]. Smart grids promise to be more resilient and survive sudden system shocks compared to their traditional grid. The smart grid promises a new type of power grid that will support decarburization and the country’s energy policy, which is desirable for sustained economic recovery post-COVID-19. The smart grid paves the way for increased use of renewable energy resources, demand response, distributed energy resources, electric vehicles, microgrids, and other smart load types. In addition, the smart grids will become intelligent power systems that offer a robust response to perturbation and rapid adaptability to dynamic changes.

### 2.1 Overview of smart grids

Because of its sophisticated nature and complex characteristics, the electric grid can be vulnerable to various security threats [1]. The very fact that the grid plays a vital role in sustaining overall societal function while being deemed an essential service, i.e., critical infrastructure, combined with common internet protocol, inherently makes the electric grid designed to be susceptible to cyber-attacks. Lastly, the distribution layer is the layer providing power predominantly to users. It transports the energy from transmission substations via high-voltage power lines to transformers, which lower the voltage for distribution along local power lines [1, 4].

In addition, the distribution layer facilitates supply transactions, i.e., prosumers (entities that consume and produce electricity) buy and sell electricity. The structure with which households and businesses get their power is known as the microgrids [1, 17]. This is the end layer of the structure and is made of distributed energy sources such as decentralized power plants and renewable energy sources. Meanwhile, the transmission layer performs long-range, high-voltage power transfers. These transfers involve power transmission from different sources, i.e., generation plants to sub-stations through transformers, which convert the incoming High-Voltage Alternating Current (HVAC) into usable power.

The typical structure of a smart grid is a multi-layered proprietary network. The three prominent layers are the generation layer, the transmission layer, and the distribution layer. The generation layer is the base layer for power generation sources, such as power plants and other renewable resources like solar and wind energy. It is made up of renewable energy sources, which are distributed all over the grid itself [1]. A smart grid is an intelligent electricity network that integrates various components and advanced technologies in an enhanced manner, including sensors, communication networks, control devices, and advanced decision-making systems. It is designed to deliver electricity more efficiently and reliably while enabling a more active role for consumers in the energy value chain. In simple terms, a smart grid is a digital energy distribution network.

### 2.2 Types of cyber attacks in smart grids

False data injection is a technique broadly agreed to by BPM as it essentially destroys the principal measurements by adding a fictitious bias in the original data and subjugating the micro PMUs’ state. The FDIAs can be distributed over pilfered or erroneous data and do not use PMU responsibilities, including traffic, which is firmly entwined with the actual PMU communications. The results, including unconventionality and observability of the data while propagating the prevailing network topology, use a covert form of corrupting the network, not raising any suspicion at the transmission in the first place. These FSK-based transmission methods can alter the original communication quite enough. They can easily hide their presence from the ESP-based screening to provide extra stealth to the attacker during transmission. The complete security of the FSK signals has ridiculed ESP as the countermeasure part due to the feasibility of the secret frequency hops. Smart grids are exposed to cybersecurity threats due to the incorporated Internet of Things (IoT) and the professional systems associated with smart grid computing technologies. The most common attacks in smart grid cybersecurity are bandwidth and routing attacks, data integrity attacks, insider and outsider threats, man-in-the-middle attacks, denial of service, replay, eavesdropping, viruses, and worms. These attacks lack confidentiality, tamper-resistance, and privacy and frequently elevate data integrity concerns.

### 2.3 False Data Injection Attacks

SGs represent a significant departure from the standard electrical power grid as they encompass the cooperation of administrators, vendors, and consumers in creating a truly interconnected system involving entities not involved in the traditional electrical power grid and administration. The extreme interconnectivity of the system raises two crucial challenges: protecting consumer privacy [18] and securing system operation [19]. Much of the threat model outlined in their research involves the real-time operation of the SG, such as events surrounding a generator synchrophasor measurement device and its use [20]. They evaluate an attack with the capacity to change the system’s state undetected as their primary focus but also briefly analyze the other practical impact of FDIAs [14].

SGs are a form of electrical power distribution grid that delivers electricity from power providers to consumers using digital communications technology to enhance system security, reliability, and economy. It differs from the conventional electrical power grid in that it enables two-way communications and monitors and controls individual pieces of the power grid. Despite their vast potential to revolutionize the electrical grid, using tech such as synchrophasor, issues around security are always cause for concern. Specifically, the FDIA [21].

The FDIA attack represents a critical cybersecurity threat to power systems, first proposed by Liu et al., where attackers compromise power system State Estimation (SE) outputs by injecting false data into meter measurements. During SE processes at control centres, Bad Data Detection (BDD) techniques are employed to spot injected false data by calculating residual vectors based on the *ℓ*_2_-norm difference between actual measurements (**y**) and estimated measurements ($\hat{\text{y}}=\text{H}\hat{\text{x}}$). Despite this, studies have shown that BDD techniques can be bypassed by FDIA attacks, where the attack-induced residual vectors fall below the BDD’s detection threshold, allowing the attack to remain undetected [14].

The construction and stealth of FDIAs involve introducing an attack vector (**a**) into the measurement data without detection. Attackers utilize different strategies, one requiring knowledge of the power system’s topology and the other, a data-driven approach known as blind FDIA. By manipulating the measurements (represented as **y**_false_ = **y** + **a**) and estimated state vectors (${\hat{\text{x}}}_{\text{false}}=\hat{\text{x}}+\text{b}$), where **b** is the error vector introduced by the attack, these attacks can remain undetectable under traditional BDD algorithms [14]. FDIA attacks often target only a few measurement devices, leading to sparse attack strategies due to attackers’ limited control or access over the system. These sparse attacks involve minimal non-zero components in the attack vector, requiring only a small number of compromised devices to launch the attack effectively.

However, FDIA frameworks have specific requirements in different application domains. For example, Wireless Sensor Networks (WSN) are more vulnerable because of their communication channels, while power systems demand a more sophisticated approach because the networks’ parameters are complicated to determine. FDIAs aim not to make the power system observable, thus requiring partial network information and few attack vectors to be efficient for the attackers, and they may be either indiscriminate or targeted based on the attacker’s strategy [22].

These malicious actors, in addition, may intend to damage the data integrity, availability, or confidentiality. For example, to forge the meter readings, which could confuse the integrity of the smart grid, hackers could either disrupt the availability of system information or breach the confidentiality of customer data, which is the core of AMIs [23]. The primary purpose of FDIAs is to overwhelm the smart grid to the point of causing transmission lines to be shut down, increasing operational expenses through the injection of false data, and significantly affecting power system operations so that the outcomes would be catastrophic for a region or a country. In a conventional unit commitment problem, it is not possible to introduce “false data” to distort the market. This is because Independent System Operators (ISOs) exist between infrastructures and markets [24]. The ISOs perform a market clearing process through the unit commitment problem. However, in an SGB, it is feasible to introduce “false data” for malicious purposes [25]. Without detecting FDIAs in an SGB, unit commitment and economic dispatch can be manipulated for various malicious actions.

## 3 Intrusion detection systems

Intrusion detection is an indispensable need for the secure operation of security tools in smart grids [7]. Intrusion detection systems are used to identify policy violations such as unauthorized access and also detect availability. In a smart grid, the nature of the network traffic may show different patterns and have unique characteristics [10]. The need for specific models having details according to the data obtained from domains is essential. Understanding the characteristics of the event logs and the domain-specific information is one of the fundamental issues for implementing an effective intrusion detection system. Most developed systems are homegrown in the literature, and most research efforts are predominantly based on VMMs (Virtual Machine Monitors). However, the availability and effectiveness of the developed detection software do not assure that intrusion detection engines can effectively detect malicious attacks in real smart grids.

Intrusion detection systems utilized in smart grids must be designed for the specific characteristics of their data, including feature selection of data [6]. With extensive data capture capabilities, deep learning classifiers have attracted attention in detecting anomalies within big datasets. This study has adapted deep learning classifiers to intrusion detection in smart grids. Specifically, two hybrid deep learning classifiers are developed, combining two robust deep learning models. These hybrid deep learning classifiers are combined with a hybrid feature selection algorithm.

### 3.1 Importance of intrusion detection systems

The original goal of utility companies, which was focused on controlling electricity demand, has been replaced by a more complex scheme where utility companies must monitor and control distributed generation and electricity demand. Consequently, to maintain control, small devices such as Remote Terminal Units (RTUs) and Intelligent Electronic Devices (IEDs) are now present in distribution systems and bear the responsibility of monitoring these systems or opening or closing circuit breakers. These devices are slowly moving towards a more complex environment due to the deployment of smart grid technologies. Despite a low-voltage level, integrating new communication services is inevitable, but developing security solutions for these devices is not progressing at the same rate.

Critical infrastructure sectors are highly dependent on the reliable and secure operation of the power grid. Therefore, distribution grid vulnerabilities can also affect these sectors in cascading and large-scale sequences. This increased dependency on dependable and safe electric power influences the importance of smart grid reliability and security. Although multiple risks are related to deploying smart grid technologies, the main security challenges are safeguarding the grid against cyber-attacks by detecting, mitigating, and responding to suspicious activities. These threats can exploit vulnerabilities in smart grid components, including home area networks, advanced metering infrastructures, distribution management systems, and microgrids.

Thus, IDS performs a different role than prevention. They protect the system from vulnerabilities that may be unwillingly opened [26]. A fragile line exists between desirable and undesirable behaviour in a system or network. A system administrator wants users to have access to their data but not anyone else’s. A system administrator wants wireless users to have web access but not to damage the network [27]. The consistent research in intrusion detection shows that the development of practical solutions to these brittle securities relies heavily on short-term solutions [26].

Intrusion detection is monitoring the events occurring in a computer system or network and analyzing them for signs of possible incidents, which are attempts to breach the system’s security policy [28]. Intrusion detection is entirely different from the traditional issues surrounding computer security. In most security approaches, the protected asset has provenance in policy and is usually an object, such as a file, a server, or a database. Access control prohibits or controls user interactions with these objects and informs the security administrator of these events. In contrast, intrusion detection focuses on the nature of the users’ interactions with the system. Protections based on historical usage are necessarily weaker than policies based on read/write/kill-or-create actions.

## 4 Feature selection techniques

It is worth mentioning that the feature selection problem can be considered a search problem, where the search space contains all features, and a search strategy is used to model the interaction among feature subsets [29]. Many meta-heuristic algorithms have been proposed to address the feature selection problem that can be numerical optimization techniques such as genetic algorithms [30]. These algorithms have also been used to solve combinatorial optimization problems like feature selection. Meta-heuristic algorithms use an iterative procedure to determine the best solution to a particular optimization problem. Namely, these algorithms search the solution space until the optimum or near-optimum solution is found.

This section briefly introduces some widely used bio-inspired techniques for feature selection for intrusion detection. These bio-inspired techniques have gained popularity in feature selection for intrusion detection due to their effectiveness and efficiency [31]. Bio-inspired techniques such as GA [32], Particle Swarm Optimization (PSO) [6], ant colony optimization (ACO), simulated annealing (SA), bee colony optimization (BCO), Artificial Bee Colony (ABC) [33], Whale Optimization Algorithm (WOA) [34], Moth-flame Optimization Algorithm (MOA) [35], and Grey Wolf Optimizer (GWO) [36] are well-known algorithms in the area of feature selection for intrusion detection.

Feature selection techniques allow for the selection of relevant features to consider in a model. This can result in more accuracy compared to models built with all dataset features. Indeed, having fewer features reduces overfitting and makes it possible to use more complex, longer time-to-train models. It also helps increase the generalization capability when new data come [6, 10]. Overall, feature selection techniques save memory and computation power and allow algorithms and machine-learning models to work faster and more efficiently. A feature extraction process is a critical step in data processing for building knowledge-based data-driven systems, which is the first stage in designing intelligent data-driven algorithms and systems. More specifically, feature selection addresses the problem of determining which input features or variables are essential for building a predictive model. It is a process in which we automatically select a subset of features relevant to the learning task. The purpose of feature selection is assumed to be threefold: building models that are easier to understand, faster to train, and more robust.

### 4.1 GWO

**Fig 2:**
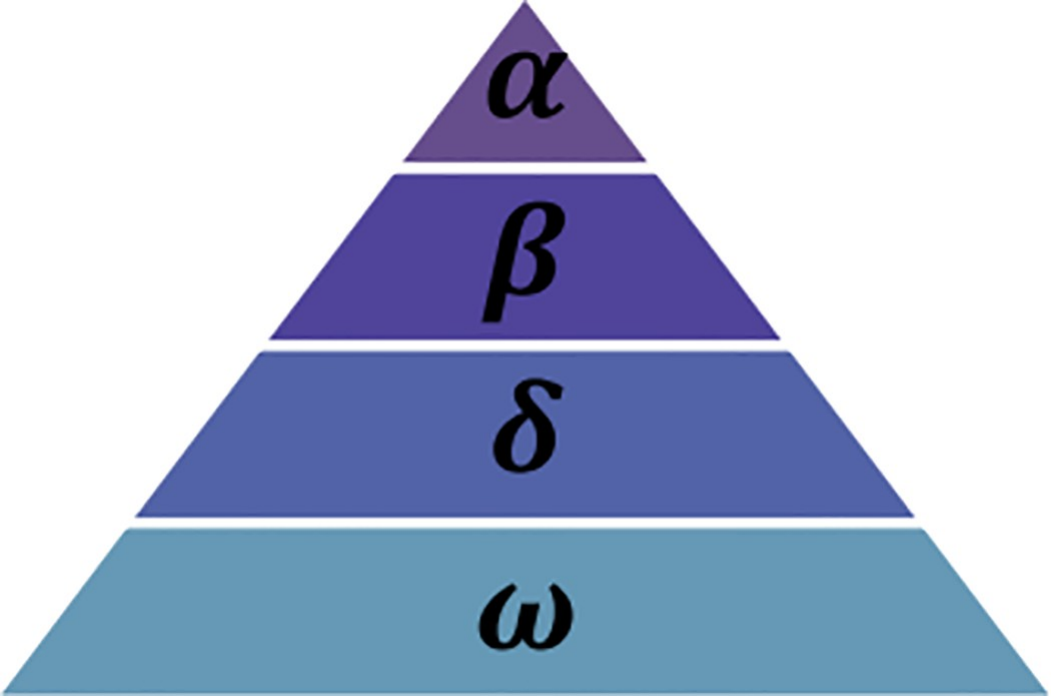
Hierarchy of Grey Wolf. This figure displays the hierarchical structure of a grey wolf pack, outlining the roles and social dynamics. (*α*) wolves leading the pack and making decisions. (*β*) wolves acting as secondary leaders and reinforcing the Alpha’s decisions. (*δ*) wolves as enforcers and messengers, maintaining order and relaying communication within the pack. (*ω*) wolves at the bottom of the hierarchy, playing a submissive role within the social order.

Grey Wolf Optimization (GWO) algorithm is a population-based metaheuristic optimization algorithm utilized to solve continuous, discrete, and mixed-variables optimization problems [37]. The most exciting component of GWO is dividing the space of the problem into only four different areas, such as alpha, beta, delta, and omega, which might lead us to the question of how these basic moves cooperate in populating the entire space of the problem [38]. The alpha (*α*), beta (*β*), delta (*δ*), and omega (*ω*) (as illustrated in Fig 2) are searching points in the grey wolf workspace, which imply that alpha is the alpha wolf of the pack, beta is the beta wolf, delta is the delta wolf, and omega is the omega wolf. The search process depends on the functions of the alpha, beta, delta, and omega representation. These are linear approximation, spherical, and circular approximation. Thus, the GWO algorithm uses the most motion of the wolves to develop the new search wave and explore the solution to the optimization problem of the workspace.

**Fig 3:**
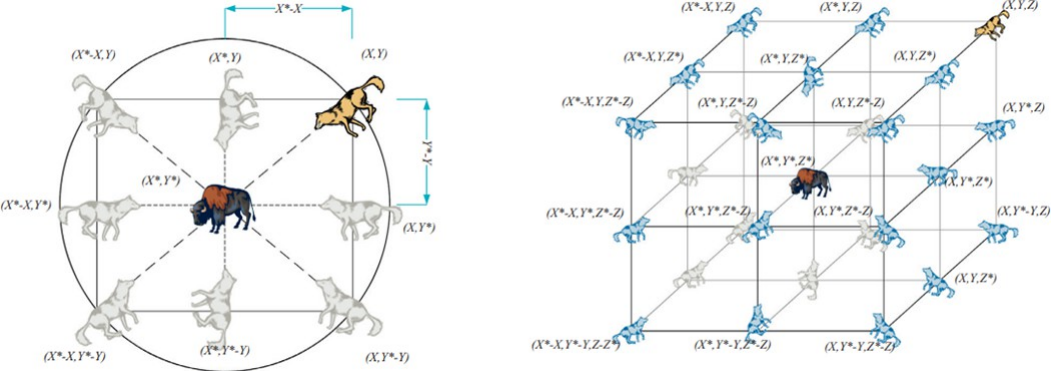
2D and 3D position vectors and their possible next locations. This figure illustrates the position vectors in two-dimensional and three-dimensional spaces and potential next locations based on movement algorithms. **2D** position vectors showing direction and magnitude in a plane. **3D** position vectors extend into spatial coordinates, providing a more complex range of movement possibilities. **Predicted next positions** calculated from current vectors, showing potential trajectories in both 2D and 3D contexts.

Fig 3 illustrates the search mechanism of the Grey Wolf Optimizer (GWO) algorithm in two dimensions. The left part of the image centres on the alpha (*α*) wolf, which leads the pack and guides the search process. Surrounding the alpha wolf are other wolves positioned at different coordinates, representing the beta (*β*) and delta (*δ*) wolves, which assist the alpha wolf in the search for prey.

The circle around the alpha wolf indicates the search radius within which other wolves (prey) are located. The notations (X, Y), (X± Y± Z), etc., denote the wolves’ positions relative to the alpha wolf, showcasing their spatial distribution in the search space. Arrows and dashed lines highlight the direction and distance between the wolves, demonstrating how they communicate and update their positions based on the alpha wolf’s guidance. The right part of the image represents an expanded version of the search mechanism, showing a three-dimensional search space. Here, the central alpha wolf is surrounded by other wolves positioned in a 3D grid, with coordinates in three dimensions (X, Y, Z). The notations (X± Y± Z), (X± Y± Z± W) indicate the wolves’ positions in the three-dimensional search space. Arrows and dashed lines continue to illustrate the interactions and positional updates between the wolves, extending the search mechanism to a more complex space.

Overall, the Grey Wolf Optimizer mimics the leadership hierarchy and hunting mechanism of grey wolves, using the positions and movements of wolves to optimize a given objective function. The leadership hierarchy involves the alpha wolf leading, followed by the beta and delta wolves, with the remaining wolves (*ω*) following. The wolves update their positions based on the (*α*), (*β*), (*δ*) wolves’ positions, simulating the hunting process. By exploring the search space in different dimensions and adjusting their positions, the wolves collectively work towards finding the optimal solution.

**Fig 4:**
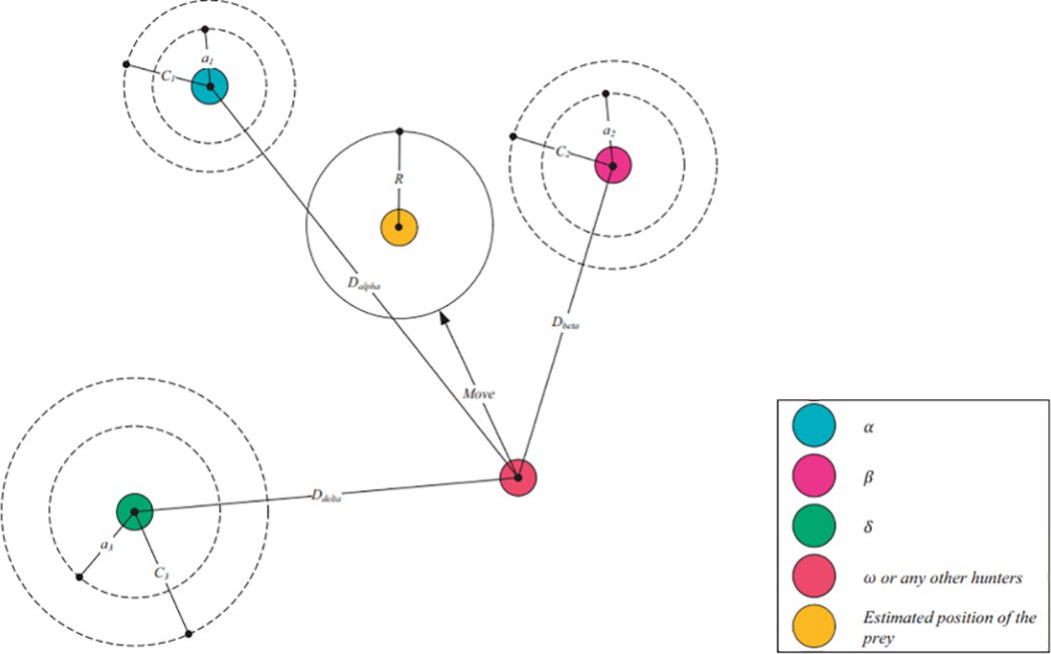
Update mechanism. This figure demonstrates the update mechanism used within the system, detailing each step in the process flow.

Fig 4 further illustrates the mechanisms of the GWO algorithm, focusing on how wolves update their positions relative to the prey during the optimization process. It shows a network of wolves around an estimated prey position, denoted by a yellow circle labelled “R.” The different types of wolves, represented by various colors, are indicated in the legend: the alpha (*α*) wolf is blue, the beta (*β*) wolf is pink, the delta (*δ*) wolf is green, the omega (*ω*) wolves or any other hunters are red, and the estimated position of the prey is yellow. The positions and movements of the wolves are key elements in the figure. The alpha (*α*) wolf is shown at position *a*_1_, surrounded by a circular search area. The beta (*β*) wolf is at position *a*_2_, also with a designated search area, and the delta (*δ*) wolf is at position *a*_3_, with its search radius marked. An omega (*ω*) wolf, or any other hunter, is shown moving towards the prey’s estimated position *R*. The distances from the omega wolf to the alpha, beta, and delta wolves are labelled *D*_*α*_, *D*_*β*_, and *D*_*δ*_, respectively. Each wolf has a dashed line indicating its search radius and the calculated distance to the prey. The prey’s position *R* serves as the target for the wolves’ movements, and the wolves update their positions by calculating the distances and directions towards *R*.

The mechanism of position update is depicted with arrows labelled “Move,” showing how each wolf adjusts its position based on the information provided by the alpha, beta, and delta wolves. Each wolf calculates its new position by considering the distance to the prey and the positions of the alpha, beta, and delta wolves. The omega (*ω*) wolf updates its position by considering *D*_*α*_, *D*_*β*_, and *D*_*δ*_, attempting to move closer to the prey’s estimated position. This collaborative approach allows the pack to effectively explore and exploit the search space, aiming to find the optimal solution.

Fig 4 also visually represents how the Grey Wolf Optimizer simulates the natural hunting strategy of grey wolves. The alpha (*α*) wolf leads the search, followed by the beta (*β*) and delta (*δ*) wolves, with the omega (*ω*) wolves adjusting their positions accordingly. The wolves use the calculated distances to the prey and the leading wolves to guide their movements. This iterative process enables the algorithm to converge on the best solution over successive iterations. In summary, the GWO’s hierarchy and position update mechanism are demonstrated, highlighting the effectiveness of this bio-inspired optimization algorithm.

The GWO has been effectively employed in feature selection to enhance IDS [39]. By mimicking the social hierarchy and hunting behaviour of grey wolves, GWO optimizes the selection of relevant features from a vast dataset, improving the accuracy and efficiency of IDS. Feature selection is crucial in reducing the dimensionality of the dataset, eliminating redundant or irrelevant features that do not contribute to intrusion detection [40]. GWO achieves this by iteratively searching the feature space and evaluating the importance of each feature based on the collective intelligence of the alpha, beta, delta, and omega wolves. This process ensures that the most significant features are selected, enhancing the system’s ability to identify and respond to potential security threats promptly.

The use of GWO in IDS improves detection accuracy and enhances computational efficiency [41]. Due to large, complex datasets processing, traditional IDS methods often suffer from high false positive rates and slow response times. GWO addresses these issues by streamlining the feature selection process, focusing on the most relevant data points [40]. This leads to faster processing times and reduced computational resources required. Moreover, the adaptability of GWO allows it to be integrated with various machine learning algorithms, further boosting the IDS’s performance. By leveraging the optimization capabilities of GWO, intrusion detection systems become more robust, capable of adapting to evolving cyber threats, and maintaining high levels of security in dynamic network environments.

### 4.2 PSO

**Fig 5:**
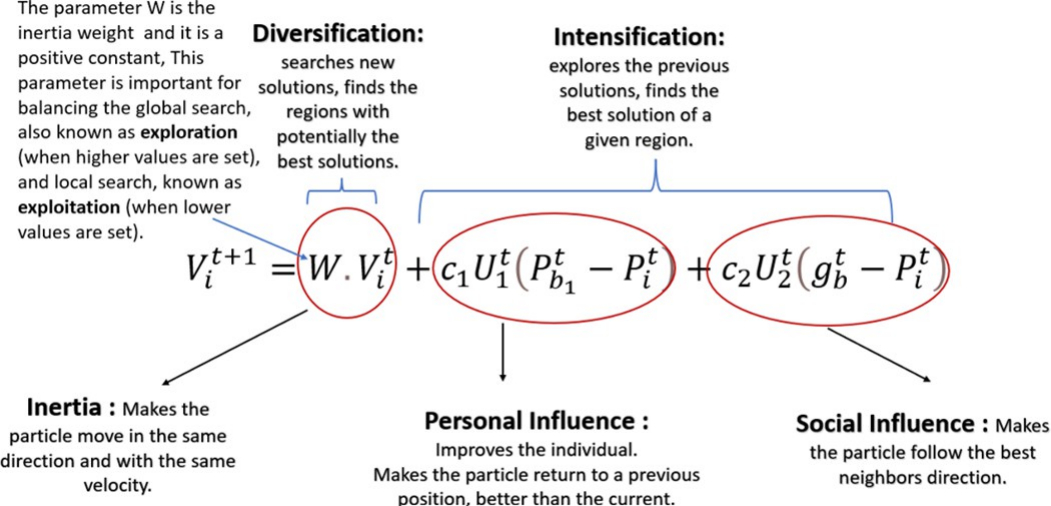
PSO mechanism. This figure illustrates the PSO process, highlighting the movement and update of particles within the solution space.

The PSO algorithm is initiated through some starting population of particles. Each particle moves quickly through the D-dimensional search area of potential solutions that depend on several factors, including global and particle best solutions and some random numbers [42]. The PSO is characterized by a swift convergence rate to the worldwide performance solution through the dynamic encouragement of the accepted population behaviour. Additionally, the PSO quickly realizes some hybridization of traditional and other metaheuristic algorithms. This would contribute to a more balanced and competitive global performance of these combined algorithms. Fig 5 depicts the velocity update equation in the PSO algorithm, highlighting the roles of different components in Eq 1:

$$\begin{array}{c} V_{i}^{t+1}=W\cdot V_{i}^{t}+C_{1}U_{1}\left(P_{i}^{t}-P_{i}^{t}\right)+C_{2}U_{2}\left(g_{b}^{t}-P_{i}^{t}\right) \end{array}$$ (1)

**Fig 6:**
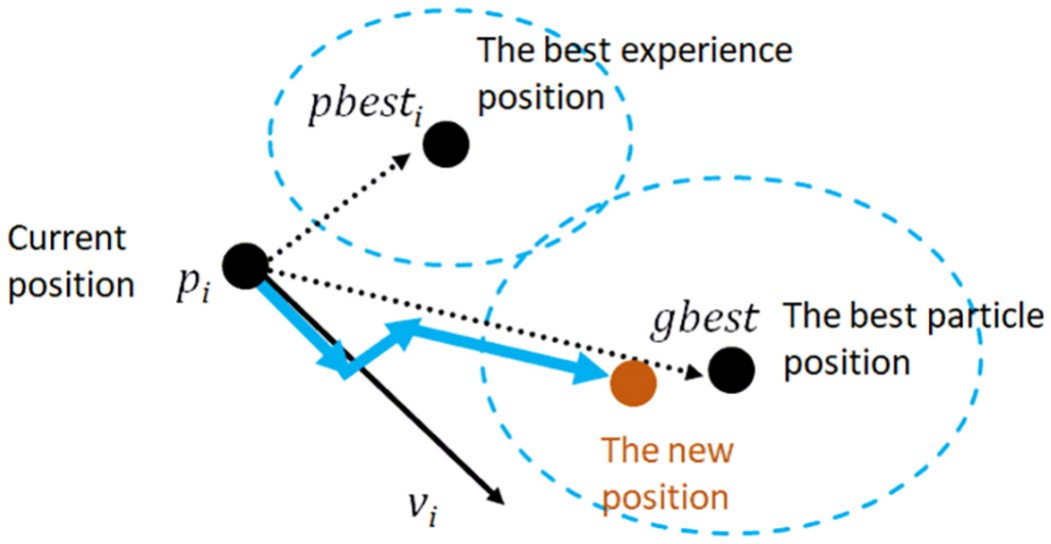
Position and velocity update in PSO. This figure illustrates the position and velocity update mechanism in PSO.

Fig 6 illustrates the position and velocity update mechanism in PSO, providing a visual representation of how each particle in the swarm adjusts its position based on its own experience and the experience of the best-performing particle. The current position of a particle is denoted by *p*_*i*_, representing where the particle is currently located in the search space. Each particle keeps track of its best position, known as the personal best position ($p_{best_{i}}$), which signifies the best solution the particle has found based on its own experience. Additionally, the global best position (*g*_*best*_) is the best position found by any particle in the swarm, representing the best solution discovered by the entire swarm. The velocity vector (*v*_*i*_) indicates the direction and magnitude of the particle’s movement, influenced by both the particle’s best and global best positions.

The position update mechanism involves the particle updating by moving towards a new position based on its current velocity. This new position combines the influences from $p_{best_{i}}$ and *g*_*best*_. The particle is attracted towards its personal best position ($p_{best_{i}}$), indicating its own best experience, and simultaneously attracted towards the global best position (*g*_*best*_), representing the best experience of the swarm. The new position is calculated by considering these influences, leading the particle to a potentially better position in the search space.

Directional arrows in the figure demonstrate the movement dynamics, with dotted lines representing the influence directions from *p*_*i*_ to $p_{best_{i}}$ and from *p*_*i*_ to *g*_*best*_. The solid blue arrows indicate the direction of the particle’s resultant movement, and the new position, shown in orange, is where the particle will move after the position update. This figure effectively demonstrates the core mechanism of PSO, where particles explore the search space by leveraging their best experiences and the collective intelligence of the swarm. Each particle adjusts its position iteratively, influenced by its personal and global best positions, enabling the swarm to converge towards an optimal solution. This balance between exploration and exploitation ensures that the particles effectively search space, leading to the discovery of high-quality solutions to optimization problems.

One of the most famous algorithms aiming to imitate the actions of a flock of birds in taking off and flying is the PSO [43]. It seeks to find the minimal value of the performance function of some problem, adjusting the fitness function in an intuitively calculated manner. If most of the flock has already reached its optimum, the algorithm becomes balanced around those best solutions. During the calculations, the individuals of the PSO population develop the same features as in other bio-inspired techniques for problem-solving: taking into account the best performance of individuals, a collective is formed, characterized by the way particles behave concerning each other. This way, the most talented birds have a predicted influence on the subsequent steps performed by the remaining birds. As with other known algorithms, the PSO diversifies global search through local neighbourhood individual bests, making it a promising method of obtaining optimal conditions.

The use of PSO in feature selection is vital for enhancing the performance of IDS [6]. Inspired by the social behaviour of bird flocking or fish schooling, PSO is a population-based optimization technique that explores and exploits the search space to identify optimal solutions. In the context of IDS, feature selection using PSO helps identify the most relevant features from a potentially large and complex dataset, which is crucial for improving the system’s detection accuracy and reducing the false positive rate. By selecting a subset of significant features, PSO ensures that the IDS can focus on the most critical aspects of the data, thereby enhancing its ability to identify and respond to malicious activities accurately. Moreover, PSO significantly contributes to the computational efficiency of intrusion detection systems. Traditional IDS methods often struggle with high-dimensional data, leading to increased processing times and resource consumption.

PSO addresses this challenge by iteratively refining the feature set, reducing the dimensionality of the data while retaining the most informative features [6]. This streamlined approach speeds up the detection process and reduces the computational load, making the IDS more efficient and responsive. Additionally, PSO’s adaptability and ease of integration with various machine learning algorithms make it a versatile tool for improving IDS performance across network environments and threat scenarios. By leveraging the optimization capabilities of PSO, intrusion detection systems can achieve higher levels of accuracy, efficiency, and adaptability, ultimately enhancing the overall security posture of the network.

## 5 Deep learning in intrusion detection

In particular, for intrusion detection in networks and computer systems, the deep feature extraction capability embedded in deep learning enables good separation and utilization of input features from both time and frequency domains. It has achieved excellent performance compared to conventional anomaly-based intrusion detection systems. Deep learning is then a natural approach to IoT security and privacy, including intrusion detection, software and hardware tamper-resistant mechanisms, secure routing, secure aggregation, secure update and status storage, secure outsourcing data operations, and secure multiparty encryption [44]. For example, deep learning mechanisms have been proposed to address network security and privacy in mobile devices and wearable technology to detect applications, malfunction URLs, auto-discover new applications, predict mobile device software vulnerabilities, notify users with on-device behaviour, and predict unusual network behaviour patterns.

One popular and successful deep learning architecture in practice is the CNN, which can be coupled with other CNN blocks and pooling layers, promoting its distinction of detecting objects and speech recognition, as well as time series and image processing, and classification of images and natural language [17, 23]. Another promising deep learning architecture is the long LSTM, which excels in recognizing patterns from sequences to capture meaningful time dependencies in a deep learning model for various sequential data. In machine learning, deep learning models are complex neural networks with at least two hidden layers (typically hundreds). It can aggregate and process many raw inputs and learn complex features. With that, deep learning has consistently shown competitive performance compared to other machine learning and computer-aided data-driven diagnosis and pattern recognition models.

### 5.1 Introduction to deep learning

Deep learning applications across a multitude of domains, including computer vision and image/video recognition and classification, have shown a variety of deep learning architectures that can be taken advantage of, such as Convolutional Neural Networks (CNN) [45], feedforward neural networks [46], Recurrent Neural Networks (RNN) [47], autoencoders [8], and generative adversarial networks [9] to name a few. Notably, previous research has performed extensive validation of deep learning methods across various classification problems, including neuroscience, music, and handwriting, from the perspective of security and forensics. Indeed, it has been demonstrated that by combining a well-thought experimental design of the deep learning model and intricate feature pooling and selection strategies, which facilitate the tuning of the network through iterated experiments, obtaining a trained classification model suited to addressing specific use cases is achievable.

In turn, these experimental designs have consistently delivered high classification performance even when the training is based on a small or medium-sized labelled dataset, alleviating data labelling costs. By leveraging these unique key advantages, deep learning neural networks have become the most favoured tools for classification.

Deep learning Neural Networks (NN) are Artificial Neural Networks (ANN) that are modelled after how the neurons operate in the human brain’s cerebral cortex [48]. Their inspiration comes from the belief that the brain’s high levels of coherence, lower error rates, fault tolerance, and multi-level instantaneous recognition are better functions that, so far, no mathematical model has been able to replicate. Indeed, deep learning neural networks have outperformed conventional machine learning methods when tackling supervised, unsupervised, and semi-supervised learning tasks. Deep learning NN demonstrates their ability to discover automatically, model, and represent the innate nature of these tasks through their deep hierarchical architectures and their training, which hinges on using collectively an extensive and labeled training set, layer-by-layer unsupervised training, and iterative fine-tuning of the network using backpropagation and error-correction procedures. Typically, these learning algorithms may stretch across many processing nodes.

### 5.2 Benefits of hybrid approaches

An enormous scope for research is handcrafted and unsupervised deep learning features. Yet deep learning tends to lose term meanings, resulting in much fewer handcrafted attributes that miss the features of importance for the machine learning approach. Semi-supervised and self-training methods were among the first hybrid models allowing the collaboration of labelled and unlabeled data. However, this paper aims to draw the benefits of human intervention to uncover the hidden data features.

Most manifold learning terms and dimensionality reduction methods have not been extensively used in anomaly detection in the smart grid; this may be due to the influence of humans on the aspect of rule technique in unsupervised learning. An exception is Isolation Forest, supported by its convolution over tree structure. It is based on random trees and is an encoder and embedded combination. Semi-supervised deep learning methods contributed to the literature. However, human-labelled data are applied in semi-supervised learning, compromising the complete data and highlighting the collaboration between SDL and the characteristics of the data to help combine labelled data with available data.

## 6 Related works (Table 1)

**Table 1:** Comparative analysis of related works.

Reference	Dataset Used	Techniques Applied	Performance Metrics	Key Limitations
[49]	10 KV solar PV system IoT data	Decision Trees, Logistic Regression, Random Forests, Isolation Forests, SVM, Autoencoders, Feed-forward Neural Networks	Decision Trees achieved the highest F1 score	Lacks a hybrid feature selection mechanism; does not address temporal dependencies in data.
[23]	IEEE 118-bus, IEEE 300-bus systems	Conditional Deep Belief Network (CDBN), ANN, SVM	Precision: 96% (CDBN)	Performance needs more improvements; lacks integration of feature selection for optimizing input features.
[50]	ICS Cyber Attack Dataset	AdaBoost, SVM, Logistic Regression, KNN, Naïve Bayes, Random Forest	Accuracy: 95% (AdaBoost)	Limited robustness against diverse FDIA scenarios; no deep learning integration for temporal analysis.
Proposed IDS	ICS Cyber Attack Dataset	PSO + GWO for feature selection, CNN-LSTM hybrid classifier	Accuracy: 97.5%, Improved F1-score	Addresses feature optimization and temporal dependency modelling; future work includes validation on additional datasets for generalizability.

The table provides a comparative analysis of related works, summarizing the datasets used, techniques applied, performance metrics, and key limitations of each study.

Smart grids, which integrate digital communication and advanced technologies for efficient and reliable power management, are susceptible to cyber threats that compromise security and functionality. The primary goal of the study conducted in [49] is to demonstrate the impact of FDIAs on power datasets and to use various machine learning algorithms to detect these attacks effectively. The researchers collected power data from a 10 KV solar PV system’s IoT server in a controlled laboratory environment to simulate and analyze FDIAs. They employed several machine learning models, including decision trees, logistic regression, random forests, isolation forests, Support Vector Machines (SVM), autoencoders, and feed-forward neural networks, to detect anomalies in the dataset. The decision tree algorithm achieved the highest performance in terms of F1 score compared with logistic regression.

An ensemble approach combining decision tree and logistic regression further improved detection performance, achieving a high F1 score and detection accuracy. The paper highlights the importance of using machine learning methods for accurate FDIA detection in smart grids. It emphasizes the advantages of ensemble techniques, which combine multiple models to enhance overall predictive accuracy and robustness. The study concludes that leveraging diverse algorithms and their complementary strengths can significantly improve the detection of cyberattacks, ensuring the security and reliability of smart grid operations.

The vulnerabilities of smart grids to cyberattacks are investigated in [23], particularly False Data Injection (FDI) attacks. The authors emphasize the importance of artificial intelligence (AI) techniques, specifically deep learning algorithms, for diagnosing these attacks in real-time using measurement data. The smart grid infrastructure, which includes AMI and SCADA systems, enables bidirectional power and data flow through advanced communication networks. Despite their advantages, smart grids face numerous potential threats and ongoing attacks. To detect unobservable FDIAs that can bypass State-Vector-Estimator (SVE) mechanisms, the authors propose a Conditional-Deep-Belief-Network (CDBN) architecture. They evaluate their detection scheme using the IEEE 118 bus and IEEE 300 bus power systems. The CDBN scheme is compared with other detection methods, such as Artificial Neural Networks (ANN) and SVM. Simulation results demonstrate that the CDBN method accurately identifies unobservable FDIAs. The study also addresses various aspects of smart grid cybersecurity, including the classification of cyberattacks based on network layers (application, transport, MAC, and physical layers), and the implementation of countermeasures such as encryption, VPN, firewalls, antivirus software, and IDS. The researchers highlight the effectiveness of deep learning techniques in maintaining the efficiency and reliability of power grids by accurately detecting and mitigating FDIAs.

In addition, Gupta et al focus on addressing the vulnerability of smart grids to cyber-attacks, specifically FDIAs [50]. The study highlights the role of AMI in monitoring and controlling power systems, which, while beneficial, also introduces new opportunities for energy theft through cyber-attacks. Integrating a cyber-layer in the metering system allows two-way communication and creates vulnerabilities that can be exploited, resulting in significant financial losses for utilities. To mitigate these risks, the authors propose a robust energy theft detection system using the AdaBoost ensemble method. This method enhances classification accuracy by sequentially creating several learning models, each correcting the misclassifications of the previous one. The proposed approach accounts for various FDIA scenarios, ensuring the system can adapt to different types of attacks.

The method effectively identifies abnormal consumption patterns indicative of energy theft by incorporating statistical and descriptive features from metering data. The performance of the proposed detection system is evaluated using the Industrial Control System (ICS) Cyber Attack Dataset (Power System Dataset) [51]. This dataset provides real-world energy consumption data, allowing for a realistic assessment of the system’s capabilities. The results demonstrate that the AdaBoost ensemble method significantly outperforms other techniques such as SVM, Logistic Regression (LR), K-Nearest Neighbors (KNN), Naïve Bayes (NB), and Random Forest Classifier (RFC). The proposed method achieves higher detection accuracy and resilience against deceptive strategies, ensuring a more reliable identification of fraudulent activities.

Smart grid cybersecurity has been extensively studied, focusing on detecting FDIAs using various methodologies. As employed by [49], traditional machine learning approaches leveraged models like Decision Trees, Logistic Regression, Random Forests, and SVMs to identify anomalies in power datasets. While these methods demonstrated effectiveness in detecting FDIAs, particularly with ensemble techniques like Decision Trees achieving high F1 scores, they lacked mechanisms for hybrid feature selection and temporal dependency analysis. The absence of these capabilities limits their ability to capture complex attack patterns and optimize input features effectively. Deep learning techniques have also been explored to enhance smart grid security. [23] proposed a CDBN architecture to detect unobservable FDIAs in real-time. Their study demonstrated the potential of deep learning for diagnosing sophisticated cyberattacks, achieving high precision on datasets like IEEE 118-bus and 300-bus systems. However, the CDBN approach is computationally intensive and does not integrate feature selection, which could further improve efficiency and accuracy. This limitation highlights the need for solutions that balance computational requirements with robust attack detection.

Existing solutions, such as those by [50], also address smart grid vulnerabilities using machine learning techniques like AdaBoost and Random Forests to detect energy theft and abnormal consumption patterns. While these approaches showed robustness in identifying deceptive attack strategies, they lacked integration of deep learning architectures that are more suited for temporal and sequential data analysis. Furthermore, their methods were not designed to handle diverse FDIA scenarios effectively. The proposed solution addresses these gaps by combining a hybrid feature selection mechanism (PSO + GWO) with a CNN-LSTM classifier, enabling feature optimization and temporal modelling. This integration enhances detection accuracy and robustness, making the system capable of addressing complex attack scenarios in smart grid environments.

## 7 Proposed IDS

**Fig 7:**
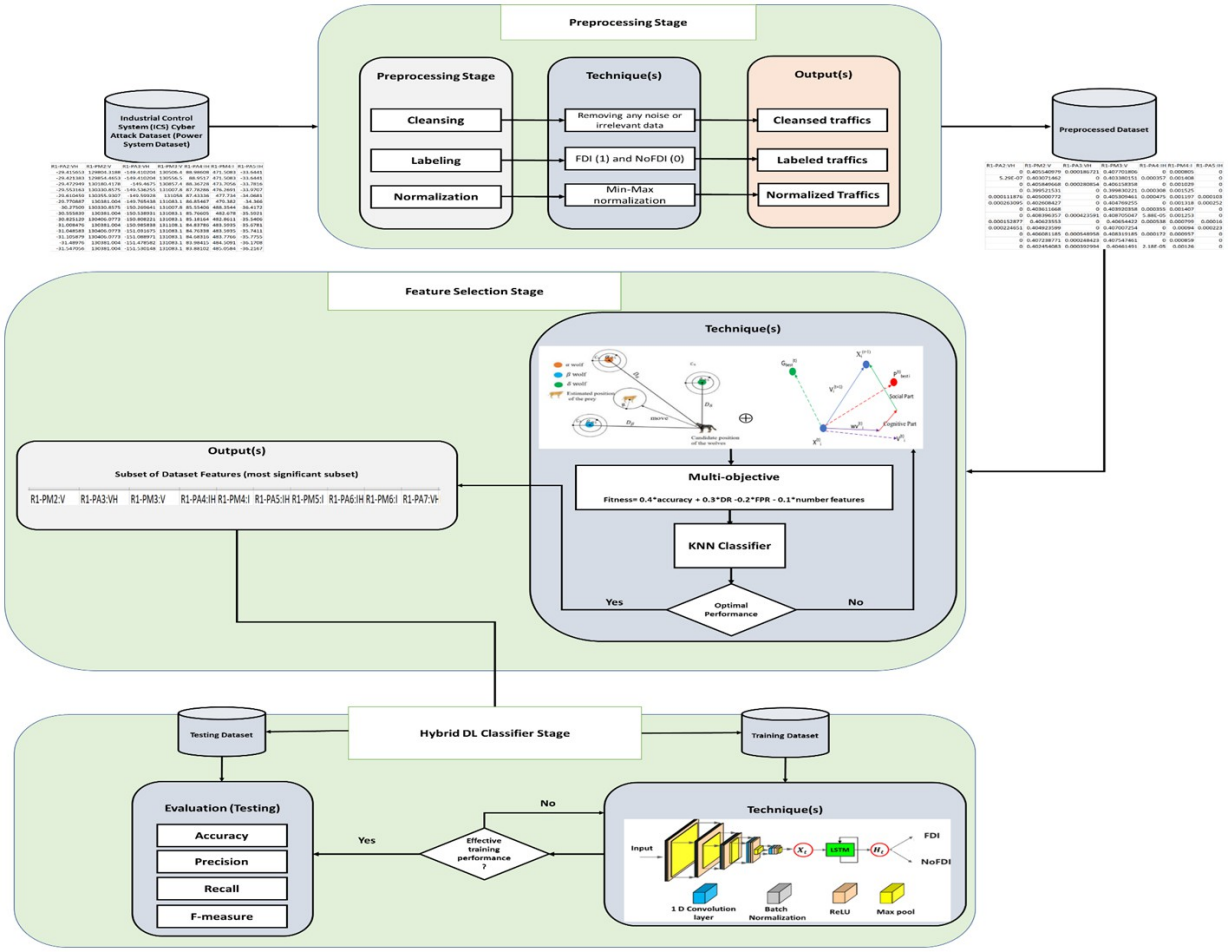
Proposed IDS architecture. This figure presents the architecture of the IDS, highlighting the primary components and data flow.

Fig 7 illustrates the overall workflow of the proposed IDS designed for FDIAs in smart grids. The process begins with the preprocessing stage, which includes cleansing, labelling, and normalizing the benchmark dataset. The preprocessed data is then subjected to a hybrid feature selection stage. PSO and GWO techniques are applied, guided by a multi-objective function to select the most relevant features. These features are then fed into the hybrid classifier stage, which integrates LSTM and CNN to capture temporal and spatial data characteristics. Finally, the testing stage evaluates the IDS’s performance based on key metrics such as accuracy, precision, recall, and F-measure using the testing dataset.

### 7.1 Preprocessing stage

This stage involves preparing the raw benchmark dataset for analysis. It includes three main steps:

#### 7.1.1 Cleansing

**Fig 8:**
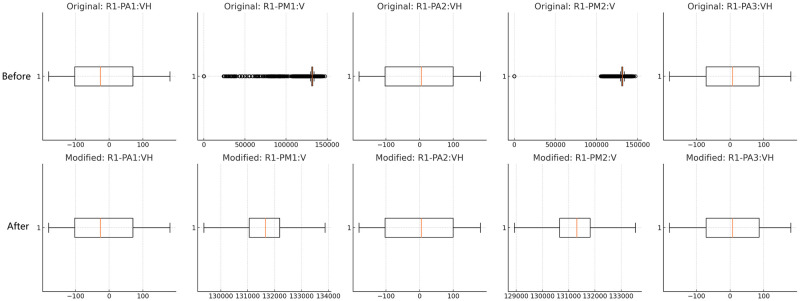
Detection and handling of outliers. This figure compares the data distributions before and after outlier handling, demonstrating reduced extreme values and improved uniformity in the modified data.

This step involves removing any noise or irrelevant data from the dataset, ensuring the data is clean and free of errors such as missing values or outliers. Cleansing is crucial for reinforcing data quality and reliability by eliminating and correcting errors in the dataset. This process addresses missing, inaccurate, noisy data that can reduce model performance. Additionally, data cleansing helps eliminate unnecessary attributes from the dataset, thereby decreasing the cost and complexity of the model [52]. Therefore, we eliminate any attribute with a null value in the benchmark dataset. Furthermore, any feature with the same value for all traffic is removed from the dataset if present. The cleansed traffic from this stage is input for the next stage (labelling). The key observations for this substage are:

- Missing Values: There doesn’t appear to be any missing data in the dataset.
- Outliers: Some columns have enormous ranges, and their standard deviations suggest possible outliers. And since the dataset is not normally distributed, the Interquartile Range—IQR test detects the outliers. The **Interquartile Range (IQR)** measures statistical dispersion, representing the range within which the central 50% of the data lies. It is calculated as:
  $$\begin{array}{c} \text{IQR}=Q_{3}-Q_{1} \end{array}$$ (2)
  Where:
  - *Q*_1_: The first quartile (25th percentile) of the data, below which 25% of the data falls.
  - *Q*_3_: The third quartile (75th percentile) of the data, below which 75% of the data falls.
  Outliers are identified as points that fall below:
  $$\begin{array}{c} Q_{1}-1.5\times \text{IQR} \end{array}$$ (3)
  or above:
  $$\begin{array}{c} Q_{3}+1.5\times \text{IQR}. \end{array}$$ (4)
  These thresholds define a “fence” for detecting extreme values:
  $$\begin{array}{c} \text{Lower}\;\text{Fence}=Q_{1}-1.5\times \text{IQR} \end{array}$$ (5)
  $$\begin{array}{c} \text{Upper}\;\text{Fence}=Q_{3}+1.5\times \text{IQR}. \end{array}$$ (6)
  Capping (Winsorization) replaces outliers with the nearest acceptable values within the range. For instance,
  - Values **below**:
    $$\begin{array}{c} Q_{1}-1.5\times \text{IQR} \end{array}$$ (7)
    are set to:
    $$\begin{array}{c} Q_{1}-1.5\times \text{IQR}. \end{array}$$ (8)
  - Values **above**:
    $$\begin{array}{c} Q_{3}+1.5\times \text{IQR} \end{array}$$ (9)
    are set to:
    $$\begin{array}{c} Q_{3}+1.5\times \text{IQR}. \end{array}$$ (10)
  This method preserves the dataset’s size while reducing the impact of extreme values. Fig 8 shows a sample of detection and handling of outliers.
- Zero Variance Columns: Some columns, such as snort_log1 through snort_log4 and control_panel_log4, have a constant value of 0 across all entries, indicating zero variance. These columns may not contribute to analysis and could be dropped if irrelevant.

#### 7.1.2 Labeling

In this step, the data is annotated with labels representing the categories or classes for the classification task. In supervised machine learning, the data is labelled, whereas in unsupervised learning, it remains unlabeled [53]. The benchmark dataset initially contains unlabeled data (classes). Thus, in this stage, the proposed approach labels the data as FDIA (1) and NoFDIA (0). The benchmark dataset is crucial for assessing detection systems, which primarily rely on labelled data (e.g., traffic). Consequently, all traffic data in the proposed dataset have been labelled as FDIA or NoFDIA, as shown in Table 2 below.

**Table 2:** Data labeling.

Symbol	Description
0	NoFDI
1	FDI

FDI refers to False Data Injection, with ‘NoFDI’ indicating no injection, while ‘FDI’ indicates the presence of false data injection within the dataset.

The labelled traffic from this stage is input for the next stage (normalization).

#### 7.1.3 Normalization

This process adjusts the data to a standard scale without distorting the differences in value ranges, thereby improving the convergence of the learning algorithm. Normalization refers to converting attribute values in a dataset to a specific range. The two primary methods of scaling are standardization and normalization. Standardization transforms attribute values based on a Gaussian distribution, while normalization scales attribute values to a standard range. Machine learning algorithms often benefit from normalization, converting dataset values without distorting the variation in their range [54, 55]. Various normalization methods exist, such as Min-Max and Z-score normalization. The proposed IDS utilizes Min-Max normalization due to its enhanced speed and learning model, which scales data between 0 and 1 according to Eq 11 [54]:

$$\begin{array}{c} X_{norm}=\frac{X+X_{min}}{X_{max}-X_{min}} \end{array}$$ (11)

In Eq 11, symbol *X* is a numerical value, *X*_*max*_, *X*_*min*_ are the maximum and minimum values of the attribute, respectively. While *X*_*norm*_ ∈ [0, 1] is a new value for the attribute.

**Fig 9:**
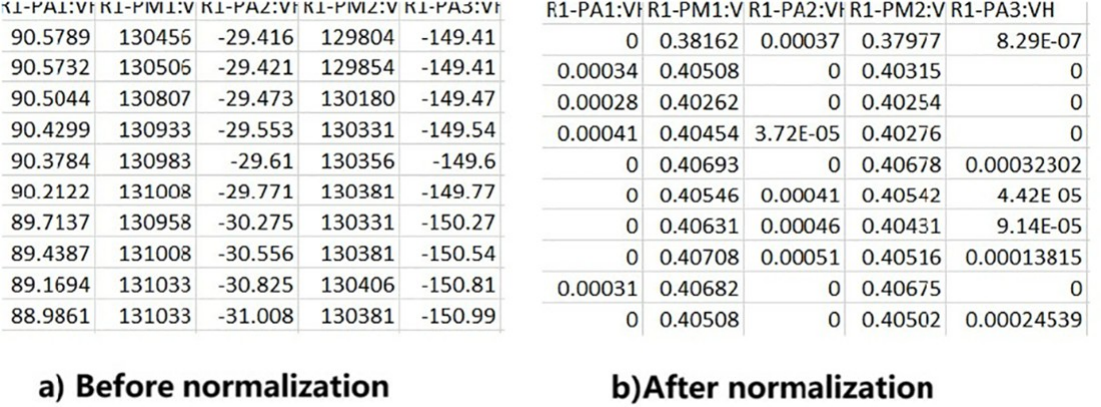
Sample of dataset before and after normalization. This figure shows a dataset sample before and after the normalization.

Fig 9 shows a sample of the dataset before and after conducting the normalization. This stage’s output is a preprocessed dataset that will be used as input for the next stage (hybrid bio-inspired feature selection mechanism).

### 7.2 Hybrid feature selection stage

This stage focuses on selecting the most relevant features from the dataset to improve the classification performance. GWO is a metaheuristic optimization algorithm inspired by the social hierarchy and hunting behaviour of grey wolves. It helps in exploring the search space efficiently to identify essential features, where the PSO is another optimization technique based on the social behaviour of birds flocking or fish schooling. It aids in finding optimal solutions by iteratively improving candidate solutions concerning a given quality measure. The motivation behind hybridizing GWO and PSO metaheuristic algorithms in the feature selection problem, particularly in attack detection, lies in leveraging the complementary strengths of both algorithms to achieve more robust and efficient solutions. Feature selection is a critical step in attack detection, as it directly impacts the detection system’s accuracy, detection rate, and false-positive rate. GWO and PSO, when combined, can provide a more balanced approach to exploring and exploiting the search space, which is essential for identifying the most relevant features for accurate attack detection.

GWO is renowned for its exploration capabilities, which help thoroughly search the solution space and avoid local optima. This characteristic is advantageous in the initial stages of feature selection, where the algorithm needs to consider a broad range of potential feature subsets. On the other hand, PSO is highly effective in exploitation, focusing on refining solutions within promising regions of the search space. This attribute is beneficial in the later stages of the search process, where fine-tuning the selected features can significantly improve detection performance. By hybridizing GWO and PSO, the resulting algorithm can effectively navigate the entire search space, combining the broad search capabilities of GWO with the fine-tuning strengths of PSO. Moreover, hybridizing these algorithms can enhance the convergence speed of the feature selection process.

PSO’s rapid convergence towards high-quality solutions can accelerate the overall search process, while GWO’s diverse search capabilities ensure that the algorithm does not prematurely converge on suboptimal solutions. This synergy is particularly valuable in attack detection, where timely and accurate feature selection is crucial for the system’s effectiveness. Additionally, the hybrid approach can reduce the number of features required for effective detection, simplifying the model and reducing computational costs. In sum, the hybridization of GWO and PSO in feature selection for attack detection aims to combine their respective strengths in exploration and exploitation, enhance convergence speed, and improve the overall quality of the selected feature set. This approach can lead to more accurate, efficient, and reliable attack detection systems, addressing the challenges posed by the complexity and variability of cyber-attacks.

The rationale behind using K-Nearest Neighbors (KNN) with feature selection and before the hybrid classifier (CNN and LSTM) lies in its role as an efficient pre-classification step that enhances the overall performance of the detection system. KNN, being a simple yet effective classification algorithm, benefits significantly from a well-optimized feature set [56–59]. The hybrid feature selection process, utilizing techniques like PSO and GWO, reduces the dimensionality of the dataset by selecting the most relevant and critical features. This step ensures that KNN works with a refined subset of data, making more accurate predictions by focusing on essential features while ignoring noise and irrelevant information. Using KNN at this stage provides a quick, reliable classification mechanism that offers an initial layer of detection by leveraging distance-based analysis, which is particularly useful when features are well-selected and optimized. KNN’s simplicity and non-parametric nature allow it to quickly highlight patterns in the optimized dataset, identifying potential anomalies or attacks with minimal computational overhead. This pre-classification step not only aids in reducing the computational burden on the more complex hybrid classifier (CNN-LSTM) but also improves the overall detection pipeline by providing an initial screening phase. The CNN-LSTM classifier, which is more resource-intensive, can then focus on further refining detection through its ability to capture spatial and temporal dependencies in the data, ensuring robust and accurate attack detection in smart grids.

Algorithm 1 represents the hybridization between GWO and PSO. As aforementioned, the hybrid GWO-PSO algorithm leverages the complementary strengths of GWO and PSO to enhance feature selection in attack detection. The initialization phase sets up the grey wolf population and particle swarm, ensuring the algorithm begins with diverse potential solutions. This diversity is crucial as it allows the algorithm to explore a wide range of feature subsets, increasing the likelihood of finding an optimal or near-optimal solution. Each entity (whether a grey wolf or a particle) is evaluated based on a fitness function, which assesses the quality of the feature subset it represents. This fitness function measures detection accuracy, false-positive rate, and computational efficiency. In the algorithm’s main loop, which runs for a predefined number of iterations, the positions of grey wolves are updated according to GWO rules. These rules mimic grey wolves’ social hierarchy and hunting behaviour, with the alpha, beta, and delta wolves guiding the rest of the pack. The positions of these wolves are adjusted to move towards the best solutions found so far, ensuring a thorough exploration of the search space. This behaviour helps avoid local optima by diversifying the search directions based on the three leading wolves.

Simultaneously, the particle swarm undergoes updates based on PSO rules. Each particle adjusts its velocity and position by considering its best-known and best-known positions of the entire swarm. This dual consideration allows particles to exploit known reasonable solutions while exploring new possibilities. The inertia weight, cognitive, and social coefficients are critical in balancing exploration and exploitation. This mechanism ensures that the swarm can quickly converge towards promising regions of the search space while still retaining the ability to explore new areas. After updating the positions of both grey wolves and particles, the algorithm evaluates the fitness of the combined population. This evaluation step is crucial as it determines which solutions are carried forward to the next generation. By selecting the best solutions from the combined population, the algorithm ensures that it continually improves the quality of the feature subsets. This selection process effectively integrates the exploration capabilities of GWO with the exploitation strengths of PSO, creating a more balanced and efficient search process.

In the final stage, the algorithm returns the optimal feature subset found during the iterations. This subset is expected to enhance the attack detection system by providing high accuracy, a high detection rate, and a low false-positive rate while maintaining computational efficiency. The hybrid GWO-PSO algorithm thus offers a robust approach to feature selection, combining the global search abilities of GWO with the local optimization strengths of PSO. This synergy makes it particularly suitable for complex problems like attack detection, where comprehensive exploration and fine-grained exploitation are essential for success.

**Algorithm 1** Hybrid GWO-PSO Algorithm for Feature Selection

Population size *N*, Maximum iterations *T* Optimal feature subset

**Step 1: Initialization**

 • Initialize the grey wolf population *X*_*i*_(*i* = 1, 2, …, *N*).

 • Initialize the particle swarm *P*_*i*_(*i* = 1, 2, …, *N*).

 • Evaluate the fitness of each grey wolf and particle.

 • Identify alpha (*α*), beta (*β*), and delta (*δ*) wolves in GWO.

 • Identify global best (*gBest*) and personal best (*pBest*_*i*_) in PSO.

**Step 2: Iterative Optimization**

**for** *t* = 1 *T* **do GWO Updates**:

 • For each grey wolf *i*, update position *X*_*i*_ using GWO update rules.

 • Evaluate fitness of updated *X*_*i*_.

 • Update *α*, *β*, and *δ* if necessary.

**PSO Updates**:

 • For each particle *i*, update velocity *v*_*i*_ and position *P*_*i*_ using PSO update rules.

 • Evaluate fitness of updated *P*_*i*_.

 • Update *gBest* and *pBest*_*i*_ if necessary.

**Combination and Selection**:

 • Combine the updated positions from GWO and PSO.

 • Evaluate the combined fitness.

 • Select the best solutions to form the next-generation population.

**Step 3: Output the Results**

 • Return the optimal feature subset.

#### 7.2.1 Multiobjective function

A multi-objective function balances different objectives, such as maximizing accuracy while minimizing the number of selected features. The proposed multi-objective function is based on the scalarization method, which combines multiple objectives into a single solution using weights. This function was incorporated into the fitness function [53, 60]. The bio-inspired algorithms GWO and PSO aim to optimize four objectives: (i) high accuracy, (ii) high detection rate, (iii) lower false-positive rate, and (iv) a smaller number of subset features, as shown in Eq 12.

$$\begin{array}{c} \begin{array}{c} Fitness=W_{1}\cdot Accuracy+W_{2}\cdot DR-\\ W_{3}\cdot FPR-W_{4}\cdot \text{number}\;\text{of}\;\text{features} \end{array} \end{array}$$ (12)

This paper employs the Rank-Sum (RS) weights method to calculate weights due to its widespread use. RS weights can be computed using Eq 13 [54].

$$\begin{array}{c} W_{i}=\frac{2(n+1-i)}{n(n+1)} \end{array}$$ (13)

Here, *W*_*i*_ represents the weight value of a variable, *n* denotes the total number of weights, and *i* is the weight number based on its order in Eq 13. Table 3 illustrates the value for each weight.

**Table 3:** Objectives weights.

Objective	Calculation	Value
*W*_1_	$W_{1}=\frac{2(4+1-1)}{4(4+1)}$	0.4
*W*_2_	$W_{2}=\frac{2(4+1-2)}{4(4+1)}$	0.3
*W*_3_	$W_{3}=\frac{2(4+1-3)}{4(4+1)}$	0.2
*W*_4_	$W_{4}=\frac{2(4+1-4)}{4(4+1)}$	0.1

Each weight (*W*_1_ to *W*_4_) is calculated using a formula that considers the position of the objective in the sequence, providing a descending weight allocation.

The output of this stage is a set of selected features that will be used for classification.

### 7.3 Hybrid classifier stage

In this stage, the selected features are fed into a hybrid classification model that combines: • LSTM: LSTM is a recurrent neural network (RNN) that is well-suited for sequence prediction problems. It can learn long-term dependencies and is effective in handling time-series data. • CNN: CNN is a class of deep neural networks commonly used for analyzing visual imagery. It is effective in capturing spatial hierarchies in data.

Hybridizing CNN and LSTM classifiers in detection, particularly for FDIAs in smart grids, offers significant advantages. After the feature selection process, combining CNN and LSTM leverages the strengths of both architectures to enhance detection performance. CNNs are adept at extracting spatial features, while LSTMs excel in capturing temporal dependencies. This hybrid approach provides a comprehensive data analysis, leading to more accurate and reliable detection of FDIs. CNNs are influential in processing grid data, especially in detecting patterns and anomalies. In the context of smart grids, CNNs can analyze the spatial features of the data, such as the distribution of power consumption across different regions or energy usage patterns. By effectively recognizing these spatial features, CNNs can identify irregularities that may indicate FDIs. This spatial analysis is crucial in understanding the local and global patterns within the grid data, which is often complex and high-dimensional.

On the other hand, LSTMs are designed to handle sequential data and are excellent at capturing temporal relationships. In smart grids, the temporal aspect is critical as power consumption and production data are inherently time-series. LSTMs can model the temporal dependencies and trends in the data, allowing anomalies to be detected over time. This capability is vital for identifying FDIs, which may not be evident from spatial features alone but become apparent when considering the temporal sequence of events. The hybrid CNN-LSTM model combines these strengths, providing a holistic data view.

After the feature selection process, where irrelevant or redundant features are removed, the remaining features are fed into the hybrid model. The CNN component first extracts spatial features from the data, which are then passed to the LSTM component, which analyzes the temporal dependencies. This integrated approach ensures that spatial and temporal anomalies are detected, leading to a more robust detection system. Moreover, hybridizing CNN and LSTM classifiers can improve the model’s generalization ability and reduce false positives. By leveraging the complementary capabilities of both architectures, the hybrid model can better distinguish between normal variations in the data and actual FDIs.

Reducing false positives is crucial for smart grid operations, as it minimizes unnecessary alerts and interventions, allowing for more efficient and effective grid management. In sum, hybridising CNN and LSTM classifiers in the detection process, particularly for FDIs in smart grids, provides a robust and comprehensive approach to anomaly detection. Combining the spatial feature extraction capabilities of CNNs with the temporal modelling strengths of LSTMs, the hybrid model can achieve higher accuracy and reliability. This approach ensures that the data’s spatial and temporal aspects are analyzed, leading to more effective detection of FDIs and enhancing smart grid systems’ overall security and stability.

As illustrated in Algorithm 2, the Hybrid CNN-LSTM for FDI Detection involves initializing the CNN and LSTM parameters, splitting the data into training and testing sets, and iteratively training the model using a combination of convolutional and LSTM layers to capture spatial and temporal features, respectively. The final classification is achieved through fully connected layers, and the model is evaluated on the testing set.

**Algorithm 2** Hybrid CNN-LSTM for FDI Detection

Selected features from the feature selection process Detection of False Data Injection (FDI)

**Step 1: Initialization**

 • Initialize the CNN and LSTM parameters.

 • Split the data into training and testing sets.

**Step 2: Training Phase training epoch CNN Component**:

 • Convolutional layers extract spatial features from the input data.

 • Apply activation functions and pooling layers to reduce dimensionality.

 • Flatten the output of the final convolutional layer.

**LSTM Component**:

 • Reshape the flattened output to match LSTM input requirements.

 • LSTM layers capture temporal dependencies in the data.

 • Apply activation functions to the LSTM outputs.

**Fully Connected Layer**:

 • Combine outputs from LSTM layers.

 • Apply fully connected (dense) layers to produce the final classification.

 • Calculate the loss and update weights using backpropagation.

**Step 3: Evaluation Phase**

 • Evaluate the model on the testing set.

**Step 4: Output**

 • Return detection results indicating FDI or no FDI.

As aforementioned, the hybrid CNN-LSTM algorithm for FDIAs detection in smart grids combines the strengths of CNNs and LSTM networks to analyze and detect anomalies effectively. The process begins with the feature selection phase, which filters out irrelevant or redundant features, leaving a refined dataset fed into the hybrid model. This ensures the model operates on the most relevant data, enhancing its efficiency and accuracy. The CNN component is responsible for extracting spatial features from the input data. CNNs are particularly effective at recognizing patterns and structures within grid data, such as the distribution and flow of power across different regions. The convolutional layers apply various filters to the input data to detect these patterns, followed by activation functions (ReLU) to introduce non-linearity and pooling layers to reduce the spatial dimensions, thereby minimizing computational complexity. The flattened output of the final convolutional layer serves as a rich feature set that encapsulates the spatial characteristics of the data.

Once the spatial features are extracted, they are reshaped to fit the input requirements of the LSTM component. LSTMs are designed to handle sequential data and are adept at capturing temporal dependencies and trends. This is crucial for smart grid data, which includes time-series information about power consumption and production. The LSTM layers process the reshaped features, learning the temporal correlations and patterns over time.

This allows the model to understand how the data evolves, which is essential for detecting anomalies that may not be apparent in static spatial data alone. The outputs from the LSTM layers are then fed into fully connected (dense) layers, which combine and refine the learned features to produce the final classification. The dense layers apply additional non-linear transformations to the data, leading to the output layer where a softmax or sigmoid activation function is used for binary or multi-class classification. This final step ensures that the model can accurately distinguish between normal and anomalous states, effectively identifying FDIs. By integrating CNNs for spatial analysis and LSTMs for temporal analysis, the hybrid model leverages the strengths of both architectures, resulting in a robust and comprehensive detection system for smart grids.

## 8 Experiments and analysis

To ensure that an IDS operates efficiently, various evaluation metrics are used to measure its performance. Common metrics include accuracy, false-positive rates, and false-negative rates. These metrics are typically derived from a confusion matrix used for two-class classifiers. The confusion matrix is a table that provides detailed information about the classification results, with each column representing instances of predicted classes and each row representing instances of actual classes. The results are summarized in Table 4. In this context, TP denotes the number of true positives, FN denotes the number of false negatives, TN denotes the number of true negatives, and FP denotes the number of false positives [53].

**Table 4:** Confusion matrix.

Actual	Predicted	
	Positive	Negative
**True**	FDI	NoFDI
	Alarms generated	No alarms
**False**	NoFDI	FDI
	Alarms generated	No alarms

The confusion matrix displays the outcomes of prediction classifications, with rows representing actual values and columns representing predicted values. ‘FDI’ and ‘NoFDI’ indicate instances of False Data Injection and no injection, respectively.

The Eqs below are used to evaluate the performance of the proposed IDS [53, 61–63]:

- **Accuracy**: The ratio of correctly predicted instances to the total instances, as shown in Eq 14.
  $$\begin{array}{c} AC=\frac{TP+TN}{TP+TN+FP+FN} \end{array}$$ (14)
- **Precision**: The ratio of true positive predictions to the sum of true positive and false positive predictions, as shown in Eq 15.
  $$\begin{array}{c} P=\frac{TP}{TP+FP} \end{array}$$ (15)
- **Recall**: The ratio of true positive predictions to the sum of true positive and false negative predictions, as shown in Eq 16.
  $$\begin{array}{c} R=\frac{TP}{TP+FN} \end{array}$$ (16)
- **F-measure**: The harmonic mean of precision and recall, providing a single metric that balances both, as shown in Eq 17.
  $$\begin{array}{c} F1=2\cdot \frac{Precision\cdot Recall}{Precision+Recall} \end{array}$$ (17)

### 8.1 Performance evaluation

The feature selection process for the GWO and PSO hybrid algorithm (GWO-PSO) is outlined in Table 5, where various parameters are presented. These include the number of wolves, particles, iterations, and the number of features selected at each optimization stage. The table highlights a range of values for each parameter, starting with 5 wolves, 20 particles, 50 iterations, and 112 features and gradually increasing to 30 wolves, 50 particles, 1000 iterations, and 32 features. This variation allows the GWO-PSO to explore and refine the feature selection process, potentially improving the efficiency and accuracy of the optimization by reducing the number of features while maintaining robust performance.

**Table 5:** Feature Selection Parameters for GWO-PSO.

No. of Wolves	No. of Particles	No. of Iterations	Number_of_Features
5	20	50	112
8	23	100	93
12	26	200	90
15	30	300	84
18	33	400	82
21	36	500	77
23	40	600	72
25	43	700	59
28	46	800	41
30	50	1000	32

This table lists the parameters used for feature selection in the GWO-PSO algorithm, including the number of wolves, particles, iterations, and selected features.

In feature selection, as presented in Table 6, reducing the number of features without compromising accuracy is a critical goal, as it leads to more efficient models with lower computational costs. The number of selected features directly impacts the performance and speed of the deep learning-based IDS, especially in real-time environments like smart grids. Selecting too many features can lead to overfitting and increased processing time, while choosing too few may result in underfitting and poor detection performance. Table 5.6 highlights the number of features selected by PSO, GWO, and the hybrid GWO-PSO method, demonstrating how the hybrid approach achieves a more balanced and efficient feature selection process. Best Feature Set: The hybrid GWO-PSO selected the smallest number of features in the best-case scenario (32 features), compared to PSO (39 features) and GWO (43 features). A smaller feature set reduces the computational load while maintaining or improving the accuracy, making the system more efficient. Worst Feature Set: Even in the worst case, the hybrid GWO-PSO algorithm selected fewer features (112 features) than GWO and PSO, which selected 129 features. This shows that the hybrid method is more efficient in reducing the feature space even in less favourable runs, contributing to lower computational costs. Standard Deviation (SD): The hybrid method also has the lowest SD (23.06), meaning it provides more consistent feature selection results across different runs, unlike GWO (27.11) and PSO (26.94), which show higher variability. Mean Number of Features: The hybrid GWO-PSO algorithm has the lowest mean number of selected features (74.2 features), compared to GWO (84.2) and PSO (81.3). This further proves that the hybrid approach is more efficient in reducing dimensionality without compromising accuracy, making it a more optimal choice for FDIA detection.

**Table 6:** Number of selected features obtained by different algorithms.

FS Algorithm	Best	Worst	SD	Mean
PSO	39	129	26.94	81.3
GWO	43	129	27.11	84.2
Hybrid GWO-PSO	32	112	23.06	74.2

This table compares the number of selected features obtained using different feature selection algorithms: PSO, GWO, and Hybrid GWO-PSO. Metrics include the best, worst, Standard Deviation (SD), and the mean number of features selected.

The performance of the hybrid GWO-PSO algorithm in feature selection is demonstrated through various metrics, including the number of wolves, particles, iterations, and selected features. As shown in Table 7, the algorithm consistently improves its fitness and accuracy as the number of iterations and particles increases. For instance, with 50 iterations, the algorithm selects 112 features with an accuracy of 35.48%, while with 1000 iterations, it selects only 32 features but achieves a much higher accuracy of 98.20%. This indicates that the hybrid GWO-PSO method effectively optimises feature selection and reduces the number of features while significantly improving the detection rate, precision, and overall performance across key metrics like recall and F-measure. The gradual reduction in the false alarm rate also suggests enhanced model reliability.

**Table 7:** Feature selection using hybrid GWO-PSO.

No. of Wolves	No. of Particles	No. of Iterations	No. of Features	Fitness	Accuracy	Detection Rate	False Alarm	Recall	Precision	F-measure
5	20	50	112	-11.04	35.48%	18.39%	37.12%	18.39%	44.26%	25.98%
8	23	100	93	-9.11	41.26%	21.70%	26.96%	21.70%	56.67%	31.38%
12	26	200	90	-8.74	55.00%	43.24%	25.53%	43.24%	73.71%	54.51%
15	30	300	84	-8.10	62.73%	51.92%	20.14%	51.92%	80.32%	63.07%
18	33	400	82	-7.84	74.40%	64.41%	12.36%	64.41%	87.36%	74.15%
21	36	500	77	-7.33	76.35%	66.50%	10.73%	66.50%	89.06%	76.15%
23	40	600	72	-6.77	88.14%	81.94%	6.39%	81.94%	91.86%	86.62%
25	43	700	59	-5.44	91.69%	88.84%	5.81%	88.84%	93.07%	90.91%
28	46	800	41	-3.63	95.04%	92.20%	2.30%	92.20%	97.40%	94.73%
30	50	1000	32	-2.71	98.20%	98.04%	1.65%	98.04%	98.20%	98.12%

This table shows the feature selection parameters and performance metrics for the Hybrid GWO-PSO algorithm, including fitness, accuracy, detection rate, false alarm rate, recall, precision, and F-measure.

**Fig 10:**
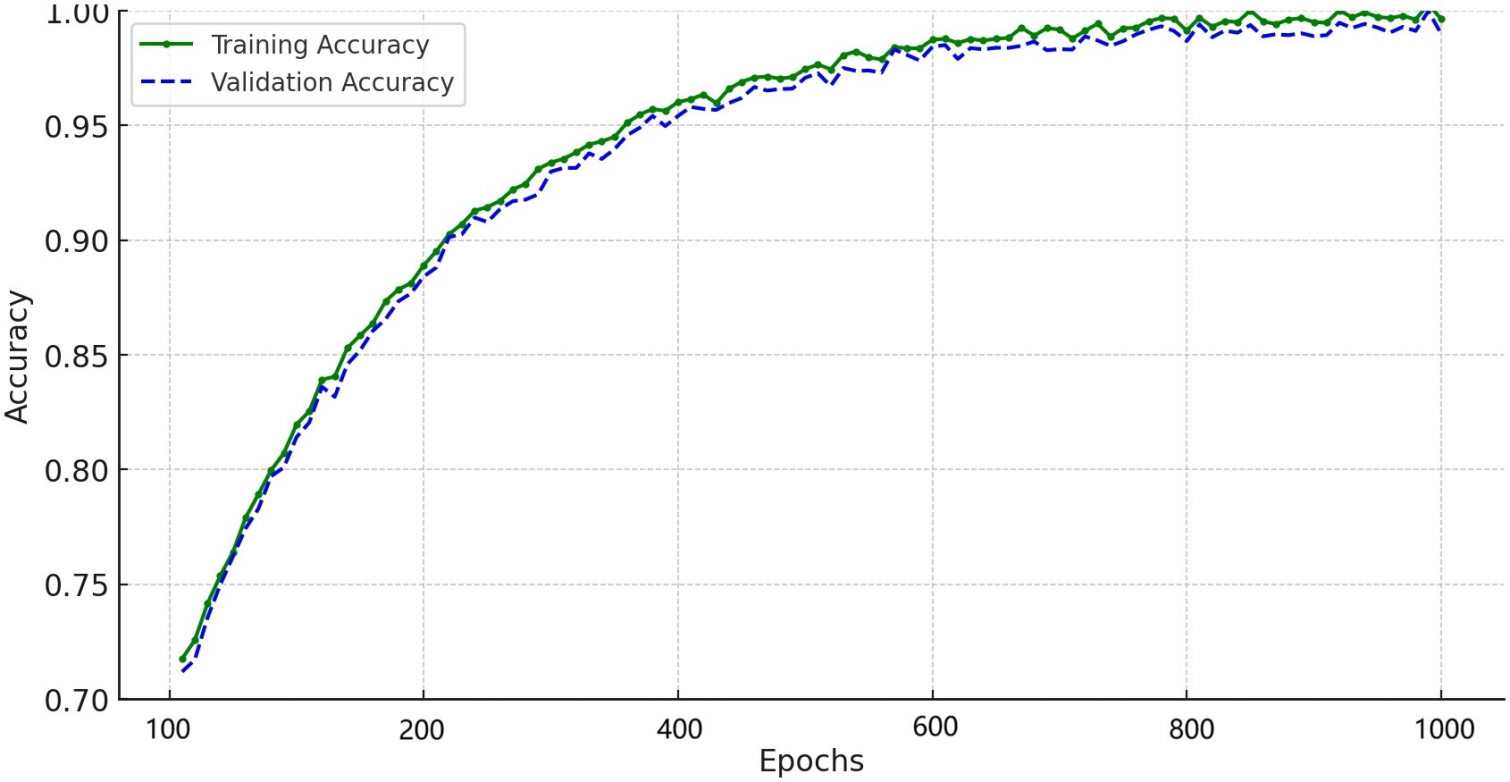
Training and validation accuracy curves. This figure presents the training and validation accuracy curves, showing the efficiency of the proposed hybrid system.

**Fig 11:**
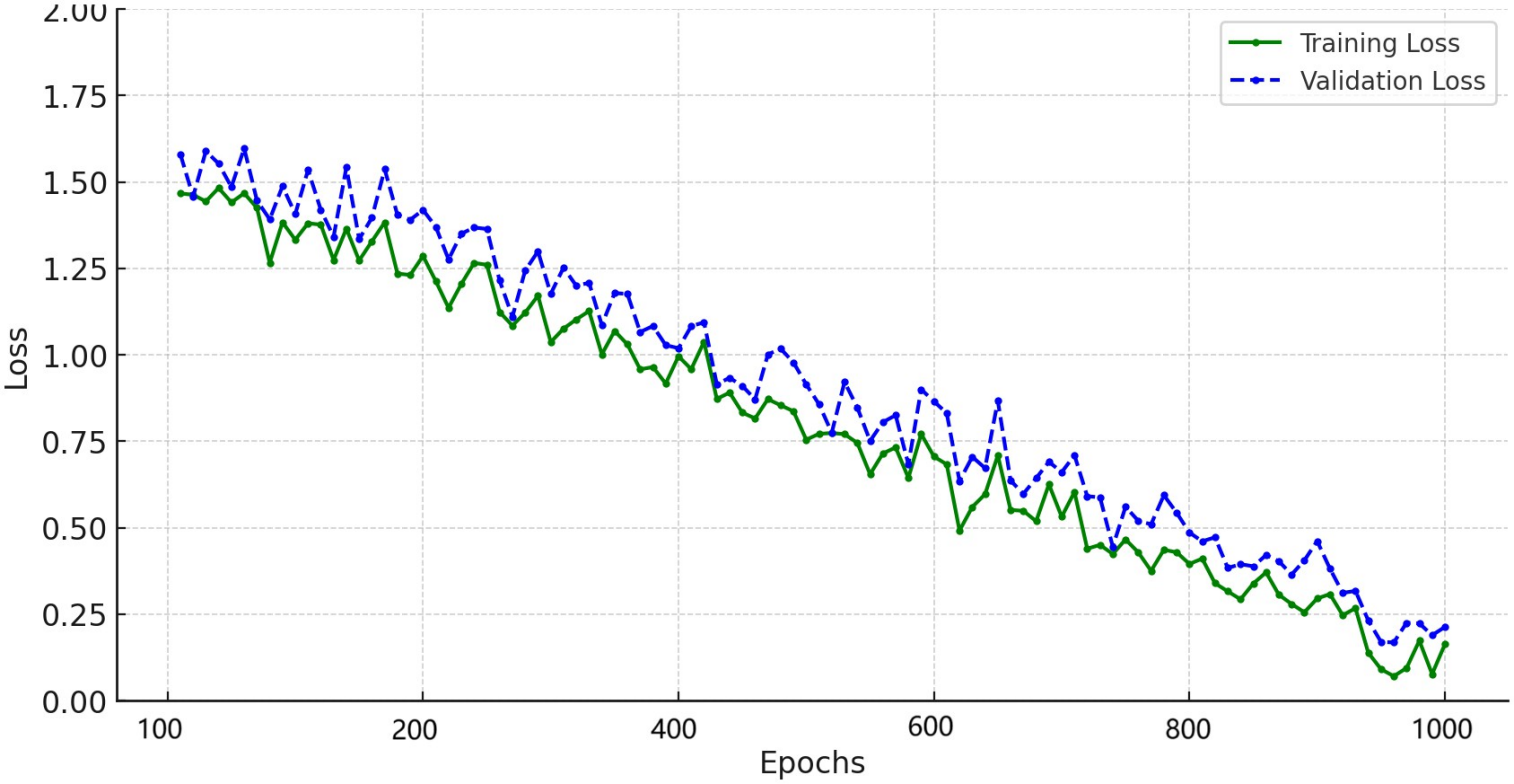
Loss function curves. This figure presents the loss function curves, showing the efficiency of the proposed hybrid system.

This section evaluates the effectiveness of the proposed IDS using the simulation results. Performance measures such as accuracy, recall, precision, and F-measure demonstrate the technique’s ability to detect FDIAs in a smart grid environment. The evaluation is implemented in Python. The provided accuracy and loss plots, in Figs 10 and 11, effectively support the performance claims of the proposed IDS for detecting FDIAs in smart grids. The accuracy plot demonstrates steady improvements in training and validation accuracy, converging towards optimal performance over 1000 epochs, reflecting the effectiveness of the hybrid CNN-LSTM classifier in learning spatial and temporal patterns. Similarly, the consistent reduction in training and validation loss highlights the model’s ability to generalize well, supported by the hybrid feature selection process using PSO and GWO optimization techniques. The minimal gap between training and validation metrics confirms that the IDS is robust, avoids overfitting, and achieves high accuracy and precision, aligning with the paper’s goal of providing a reliable and computationally efficient detection system.

The proposed IDS is compared to traditional methods, including RNN, CNN, DNN, and other state-of-the-art IDSs. The goal is to assess its effectiveness in detecting FDIAs within smart grids. The hyperparameters of the hybrid deep learning models are carefully selected based on extensive experiments, and the parameters for bioinspired feature selection algorithms are also fine-tuned. In the smart grid benchmark dataset, the proposed IDS effectively detects FDIAs, mitigating their negative impact on smart grids. Tables 8–11 list the implementation parameters of the technique.

**Table 8:** CNN-LSTM parameters.

Parameter	Value
Random Seed (numpy)	42
Random Seed (tensorflow)	42
Number of Classes	2
Test Size	0.325
Filters (Conv1D)	32
Kernel Size (Conv1D)	2
Dropout Rate (1st layer)	0.2
Pool Size (MaxPooling1D)	2
LSTM Units	50
Dense Units (1st layer)	128
Dropout Rate (2nd layer)	0.5
Dense Units (2nd layer)	64
Dense Units (Output)	Number of Classes (2)
Activation Function	Softmax
Loss Function	Sparse Categorical Crossentropy
Optimizer	Adam
Epochs	100
Batch Size	64

This table presents the parameters used in the CNN-LSTM model, including configuration details for the layers, activation function, and optimization.

**Table 9:** GWO parameters.

Parameter	Value
K-value (KNN)	5
Number of Wolves	10
Maximum Iterations	100
Train-Test Split	80% Train, 20% Test

This table presents the parameters for GWO, including settings for K-value in KNN, the number of wolves, maximum iterations, and train-test split ratio.

**Table 10:** Hybrid bio-inspired feature selection.

Parameter	Value
K-value (KNN)	5
Number of Particles	10
Maximum Iterations	100
Train-Test Split	80% Train, 20% Test

This table outlines the parameters for the hybrid bio-inspired feature selection method, including K-value for KNN, number of particles, maximum iterations, and train-test split ratio.

**Table 11:** PSO parameters.

Parameter	Value
K-value (KNN)	5
Number of Particles	10
Maximum Iterations	10
Train-Test Split	80% Train, 20% Test

This table presents the parameters for the PSO algorithm, including the K-value for KNN, the number of particles, maximum iterations, and the train-test split ratio.

**Fig 12:**
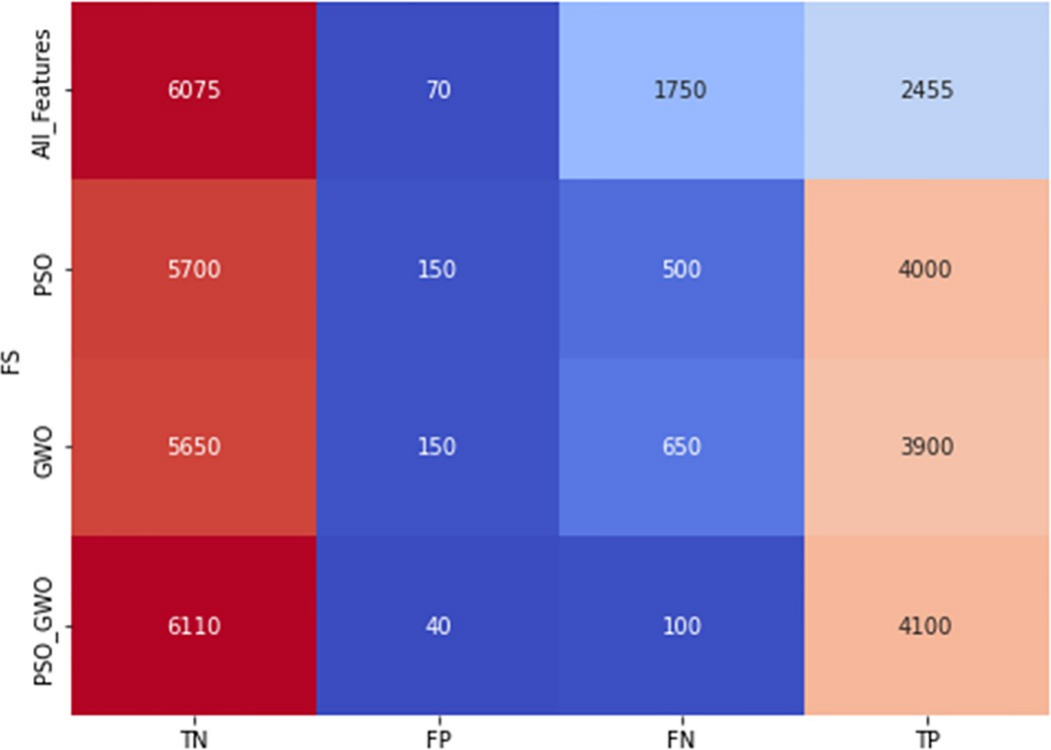
Heatmap of feature correlations. This heatmap visualizes the correlation between different features within the dataset, providing insights into feature relationships.

Fig 12 visualizes the performance of the hybrid CNN-LSTM model based on various feature selection techniques: All Features, PSO, GWO, and PSO_GWO. The TN, FP, FN, and TP are compared across these feature sets to assess the model’s effectiveness in detecting False Data Injection Attacks (FDIAs) in smart grids. The “All_Features” set includes all possible features without any selection optimization, resulting in moderate TN and TP values. The high FN count suggests that this approach may miss many actual attacks, leading to a lower detection accuracy. However, the FP rate is relatively low, indicating fewer false alarms. This balance suggests that while the model can identify attacks with some accuracy, it may not be the most efficient due to the inclusion of irrelevant or redundant features, which can hinder performance.

The PSO and GWO feature selection techniques significantly improve the model’s performance. Both methods reduce FN counts compared to the All_Features set, indicating a higher true positive rate and better detection accuracy. PSO achieves a higher TP count than GWO, demonstrating its effectiveness in capturing more true attack instances. However, GWO has a higher TN count, suggesting it is better at correctly identifying non-attack instances. The combination of PSO and GWO (PSO_GWO) further enhances the model’s performance, achieving the highest TP count and the lowest FN count. This indicates a superior detection capability with minimal missed attacks, validating the effectiveness of hybrid feature selection in optimizing the model’s performance.

The overall analysis demonstrates that the hybrid CNN-LSTM model, when combined with advanced feature selection techniques like PSO and GWO, significantly improves the detection accuracy of FDIAs in smart grids. The PSO_GWO combination offers the best performance, highlighting the importance of optimizing feature selection to enhance the robustness and reliability of intrusion detection systems. This hybrid approach ensures a comprehensive analysis by leveraging the strengths of both spatial and temporal data features, ultimately leading to a more secure and resilient smart grid infrastructure.

Table 12 illustrates the performance of the hybrid CNN-LSTM model using various feature selection techniques—All Features, PSO, GWO, and PSO_GWO—across different epochs (10, 50, 100, 200, 500, 1000). The metrics evaluated include Accuracy, False Alarm, Recall, Precision, and F-measure. These metrics provide a comprehensive view of how effectively the model can detect False Data Injection Attacks (FDIAs) in smart grids.

**Table 12:** Performance of hybrid CNN-LSTM model with various feature selection techniques.

Epoch	Features	Accuracy	False Alarm	Recall	Precision	F-measure
10	All_Features	52.03%	10.79%	20.19%	68.62%	31.20%
	PSO	53.14%	8.40%	20.39%	74.03%	31.98%
	GWO	53.09%	8.68%	21.72%	75.30%	33.72%
	PSO_GWO	55.89%	9.26%	24.38%	74.44%	36.73%
50	All_Features	56.78%	7.23%	24.27%	78.81%	37.12%
	PSO	57.07%	6.94%	27.35%	82.67%	41.10%
	GWO	55.82%	7.38%	23.84%	78.81%	36.60%
	PSO_GWO	57.29%	6.58%	28.84%	84.77%	43.04%
100	All_Features	59.90%	6.82%	27.20%	80.23%	40.63%
	PSO	60.39%	5.88%	27.62%	82.86%	41.43%
	GWO	59.90%	5.79%	25.53%	81.48%	38.88%
	PSO_GWO	64.73%	4.69%	32.27%	86.63%	47.02%
200	All_Features	69.08%	3.77%	40.59%	91.11%	56.16%
	PSO	71.01%	3.45%	38.46%	89.74%	53.85%
	GWO	70.05%	3.51%	37.63%	89.74%	53.03%
	PSO_GWO	76.33%	2.52%	47.73%	93.33%	63.16%
500	All_Features	81.64%	1.63%	57.14%	96.00%	71.64%
	PSO	86.96%	2.26%	67.57%	94.34%	78.74%
	GWO	88.89%	2.19%	71.43%	94.34%	81.30%
	PSO_GWO	90.92%	1.96%	75.00%	94.49%	83.62%
1000	All_Features	82.42%	1.14%	58.38%	97.23%	72.96%
	PSO	93.72%	2.56%	88.89%	96.39%	92.49%
	GWO	92.27%	2.59%	85.71%	96.30%	90.70%
	PSO_GWO	98.65%	0.65%	97.62%	99.03%	98.32%

This table presents the performance metrics of the hybrid CNN-LSTM model across various feature selection techniques, including PSO, GWO, and their hybrid (PSO_GWO), for different epochs. Metrics include accuracy, false alarm rate, recall, precision, and F-measure.

Initially, at 10 epochs, the performance metrics for all feature selection techniques show moderate values. The PSO_GWO combination yields the highest accuracy (55.89%), recall (24.38%), and F-measure (36.73%), indicating its superiority in identifying true positives and maintaining a balance between precision and recall. However, the false alarm rate is slightly higher than PSO and GWO individually. This trend indicates that while the hybrid approach captures more attacks, it also introduces some false positives, highlighting the trade-off between detection rate and false alarms.

As the number of epochs increases, significant improvements are observed across all metrics. By 200 epochs, PSO_GWO consistently outperforms the other techniques, achieving an accuracy of 76.33%, a recall of 47.73%, and an F-measure of 63.16%. This indicates a robust enhancement in the model’s ability to detect FDIAs while minimizing false positives. The hybrid approach’s ability to combine PSO’s and GWO strengths ensures that the most relevant features are selected, leading to better model training and more accurate predictions. The false alarm rate for PSO_GWO also drops significantly to 2.52%, reinforcing its effectiveness in distinguishing between legitimate and malicious data.

**Fig 13:**
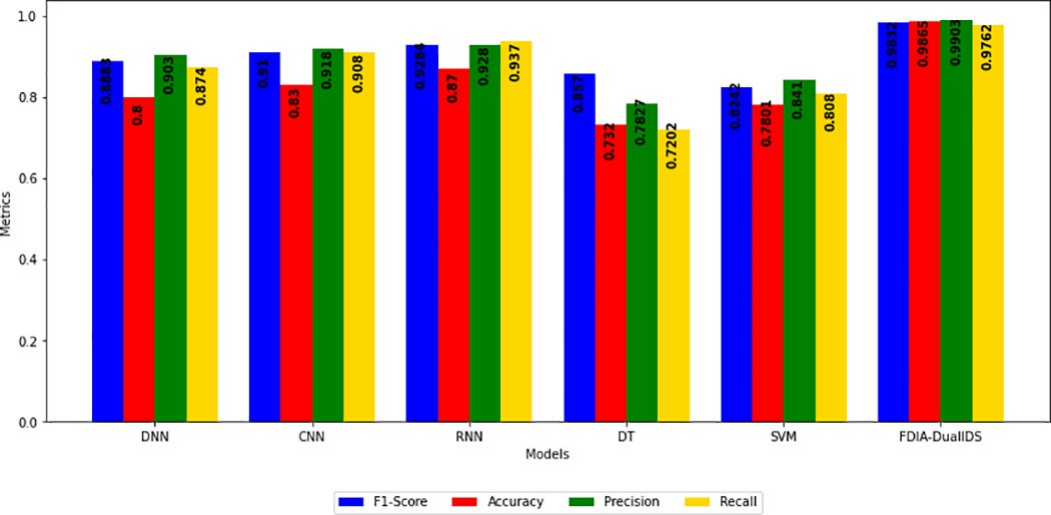
Comparison with state-of-the-art IDSs. This figure compares the proposed IDS with various state-of-the-art IDS solutions, showcasing performance metrics and evaluation results.

Fig 13 showcases the performance of various machine learning models in detecting FDIAs in smart grids, evaluated based on key metrics: F1-Score, Accuracy, Precision, and Recall. The models compared include DNN, CNN, RNN, Decision Tree (DT), Support Vector Machine (SVM), and the proposed IDS model, FDIA-DualIDS. It is evident from the visual representation that the FDIA-DualIDS outperforms the other models across all metrics, showcasing its effectiveness and robustness in FDIA detection.

Accuracy, representing the overall correctness of the model, is another critical metric. The FDIA-DualIDS model demonstrates an accuracy of 0.9865, substantially higher than the other models, with the closest competitor being the RNN model at 0.8700. This high accuracy indicates that the FDIA-DualIDS model correctly classifies a more significant proportion of the data, making it a more dependable choice for practical implementation in smart grids. The enhanced accuracy is critical in real-world applications where false alarms can lead to unnecessary interventions and operational inefficiencies. Precision, the proportion of true positive predictions among all positive predictions, is also notably high for the FDIA-DualIDS model, at 0.9903. This indicates that the model has a very low false positive rate, ensuring that almost all identified attacks are actual threats. This level of precision is critical for smart grid systems, where false positives can disrupt normal operations and lead to unnecessary corrective actions. The precision of the FDIA-DualIDS model far surpasses that of the other models, highlighting its effectiveness in distinguishing between normal operations and potential threats.

Recall that the proportion of actual positive cases correctly identified is another area where the FDIA-DualIDS excels, achieving a value of 0.9762. High recall is essential in ensuring that the model captures the majority of true attacks, minimizing the risk of undetected threats that could compromise the grid’s security. The other models show lower recall values, indicating a higher likelihood of missing actual attacks. The FDIA-DualIDS model’s high recall rate demonstrates its ability to detect and respond to FDIAs, ensuring robust protection for smart grid systems.

The F1-Score, the harmonic mean of precision and recall, indicates the balance between the two metrics. The FDIA-DualIDS model achieves an F1-Score of 0.9832, significantly higher than the other models, whose F1-Scores range from 0.8242 to 0.9284. This high F1-Score reflects the model’s ability to accurately identify true positives while minimizing false negatives, making it highly reliable in identifying FDIAs. The superior F1-Score of FDIA-DualIDS underscores its enhanced capability in handling the complexities of smart grid data, where both precision and recall are crucial for maintaining grid stability and security.

In summary, the proposed FDIA-DualIDS model exhibits superior performance across all key metrics compared to traditional models like DNN, CNN, RNN, DT, and SVM. Its high F1-Score, accuracy, precision, and recall make it an excellent choice for detecting FDIAs in smart grids. The FDIA-DualIDS model’s advanced feature selection and hybrid deep learning approach enable it to effectively handle the complexities of smart grid data, ensuring reliable and efficient threat detection. This performance highlights the potential of the FDIA-DualIDS model to significantly enhance the security and reliability of smart grid systems, paving the way for its adoption in real-world applications.

In addition, Table 13 provides a comparative analysis of various state-of-the-art IDSs based on key performance metrics such as accuracy, false alarm rate, recall, precision, and F-measure. The analysis covers a range of IDS techniques, including XGBoost, AdaBoost, A-BiTG [64], AT-GVAE [65], DAMGAT [66], and the proposed DeepSelect-FDIA method. Each IDS demonstrates varying degrees of efficiency across the metrics, highlighting the strengths and weaknesses of each approach. For example, XGBoost achieves an accuracy of 87.83%, with a false alarm rate of 10.33%, while A-BiTG improves accuracy to 93.72% and reduces the false alarm rate to 5.45%. The proposed DeepSelect-FDIA method outperforms all the compared IDS techniques, as shown in Table 13. It achieves an outstanding accuracy of 98.65%, significantly higher than the other IDSs, and the lowest false alarm rate at only 0.65%. The recall, precision, and F-measure values for DeepSelect-FDIA are also notably superior, with 97.62% recall, 99.03% precision, and an F-measure of 98.32%. This indicates that the proposed method not only excels in detecting intrusions with high precision but also maintains a minimal false alarm rate, making it a highly reliable solution for detecting FDIAs. In comparison, DAMGAT achieves an accuracy of 94.88 with a false alarm rate of 3.35, making it the closest competitor to the proposed DeepSelect-FDIA in terms of accuracy. However, its recall, precision, and F-measure values are still lower than DeepSelect-FDIA, at 92.16%, 94.71%, and 93.42%, respectively. These results demonstrate the superior performance of the DeepSelect-FDIA, particularly in maintaining a balance between high accuracy and low false alarms, which is crucial for effective and reliable intrusion detection in complex environments.

**Table 13:** Comparison with state-of-the-art IDSs.

IDS	Accuracy	False Alarm	Recall	Precision	F-measure
XGBoost	87.83%	10.33%	85.18%	85.18%	85.18%
AdaBoost	88.12%	10.40%	86.01%	85.42%	85.71%
A-BiTG [64]	93.72%	5.45%	92.46%	91.79%	92.12%
AT-GVAE [65]	93.53%	4.79%	90.95%	92.54%	91.74%
DAMGAT [66]	94.88%	3.35%	92.16%	94.71%	93.42%
**DeepSelect-FDIA (Proposed)**	**98.65%**	**0.65%**	**97.62%**	**99.03%**	**98.32%**

This table compares the proposed DeepSelect-FDIA IDS with various state-of-the-art IDSs, presenting metrics such as accuracy, false alarm rate, recall, precision, and F-measure.

### 8.2 Analysis and discussion

Hybrid feature selection methods, specifically PSO and GWO, are crucial in detecting FDIAs in smart grids. These bio-inspired algorithms effectively navigate the search space to identify the most relevant features for accurate attack detection. PSO excels in exploration, rapidly converging towards optimal solutions, while GWO’s social hierarchy and hunting behaviour ensure a thorough search, avoiding local optima. This complementary synergy results in a refined feature subset that significantly enhances the performance of the IDS.nA multi-objective function plays a pivotal role in balancing various critical criteria such as accuracy, recall, precision, and the number of features. By combining multiple objectives into a single optimization problem with a scalarization method, the multi-objective function ensures that the selected features maximize detection performance while minimizing computational complexity. This balanced approach allows the IDS to operate efficiently, maintaining high accuracy and detection rates without compromising on speed and resource utilization. This is essential for real-time smart grid applications.

Hybrid deep learning classifiers, combining CNN and LSTM networks, offer a robust mechanism for detecting FDIAs. CNNs excel in extracting spatial features from smart grid data and identifying patterns and anomalies in the distribution of power usage. On the other hand, LSTMs are adept at capturing temporal dependencies, which are crucial for analyzing time-series data inherent in smart grids. Integrating these two architectures ensures that spatial and temporal characteristics are effectively investigated, leading to more comprehensive and accurate detection of anomalies. The impact of these methodologies on accuracy is profound. The hybrid feature selection ensures that only the most relevant features are used, reducing noise and irrelevant data that could otherwise lower accuracy. The multi-objective function refines this process by balancing the trade-offs between various performance metrics.

The hybrid CNN-LSTM classifier then leverages these refined features to achieve high accuracy, consistently identifying true positives and minimizing false negatives, which is critical for maintaining the reliability of smart grid operations. In addition, recall that the measure of the IDS’s ability to identify all relevant instances of FDIAs is significantly enhanced through these combined approaches. The hybrid feature selection ensures that critical features applicable to all types of attacks are included. In contrast, the hybrid deep learning classifier’s ability to understand complex patterns from spatial and temporal data ensures that even subtle anomalies are detected. This comprehensive detection capability minimizes the chances of undetected attacks, ensuring the safety and stability of the smart grid.

Precision, which measures the proportion of true positive detections among all positive detections, is also improved. The refined feature set provided by the hybrid feature selection reduces the chances of false positives, as irrelevant features that could trigger false alarms are eliminated. The hybrid deep learning classifier’s sophisticated analysis further ensures that detected anomalies are indeed indicative of actual attacks, thereby increasing precision. Reducing false positives is crucial for preventing unnecessary interventions and maintaining operational efficiency. Besides, the F-measure, which harmonizes recall and precision, benefits from the improvements in both metrics. A high F-measure indicates that the IDS is both sensitive (high recall) and specific (high precision). The hybrid methodologies ensure that the IDS does not compromise on either aspect, providing a balanced and effective detection mechanism. This balanced performance is vital for smart grids, where both the detection of all potential threats and the minimization of false alarms are equally important.

In sum, combining hybrid feature selection, a multi-objective function, and hybrid deep learning classifiers creates a powerful IDS framework for detecting FDIAs in smart grids. Each component is critical in enhancing performance metrics, resulting in a superior detection system. This comprehensive approach ensures that the IDS operates with high accuracy, recall, precision, and F-measure, effectively securing smart grids against complex and evolving cyber threats.

## 9 Conclusion

This paper proposes a novel IDS framework that detects FDIAs in smart grids. By integrating feature selection methods with hybrid deep learning classifiers, we achieved substantial improvements in detection accuracy and robustness over traditional IDS approaches. This dual-hybrid approach harnesses the strengths of PSO and GWO for efficient feature selection, paired with the combined capabilities of CNN and LSTM networks for comprehensive data classification. Experimental results on benchmark datasets demonstrate the effectiveness of the proposed method in bolstering the security and reliability of smart grid systems, underscoring its potential for deployment in critical infrastructure. The findings have important implications for science and technology policies, especially in promoting the integration of advanced, AI-driven security measures within national energy infrastructures. As smart grids become a cornerstone of energy systems, policymakers must adopt frameworks that support adaptive, machine learning-based cybersecurity solutions. Such solutions can improve detection accuracy and operational resilience, providing a science-backed foundation for more stringent cybersecurity standards. This paper also highlights the role of advanced IDS frameworks in accelerating the adoption of other innovative technologies within the energy sector. Deploying robust IDS models like the proposed dual-hybrid approach can mitigate cyber risks and support the safe integration of smart devices, such as automated control systems and distributed energy resources. By securing these technologies, the energy sector can benefit from more intelligent, autonomous operational capabilities, ultimately driving progress toward secure and resilient smart grids. Future research should incorporate real-world smart grid data and additional datasets from different smart grid environments and configurations to further validate and refine the IDS framework, expanding its applicability to other cyber-physical systems beyond smart grids. Developing adaptive learning mechanisms that dynamically update IDS models in response to evolving threats would enhance the system’s resilience. Investigating other bio-inspired optimization algorithms and advanced deep learning architectures may further optimize detection performance. Finally, addressing real-time processing and scalability challenges in large-scale smart grid environments will be essential for practical implementation and widespread adoption of advanced IDS solutions in energy infrastructures.
