# Expression of Concern: Dual-hybrid intrusion detection system to detect False Data Injection in smart grids

**DOI:** 10.1371/journal.pone.0344254

**Published:** 2026-03-05

**Authors:** The PLOS One Editors

After this article [1] was published, concerns were raised about the following:

- Compliance with the PLOS Authorship policy;
- [1] appears to report a similar study and methodology as an article published later by the same author group [2],
- The article’s peer review.

In response to editorial follow-up, first author SHM stated that, though [1] and [2] share elements, the reported studies differ in analytical focus, architecture, methodological intervention, and results.

In light of the cumulative issues and concerns which were not resolved in our discussions with the authors, the *PLOS One* Editors issue this Expression of Concern. Readers are advised to interpret the article [1] with caution.
